# Targeted Metabolomics of *Tityus* Scorpion Venoms: Unveiling Small-Molecule Components

**DOI:** 10.1021/jasms.5c00238

**Published:** 2025-10-07

**Authors:** Nathalia Baptista Dias, Bibiana Monson de Souza, Geovanny Barroso, Javier Ortiz Leiva, Gabriela Mendonça Paula, Hipócrates M. Chalkidis, Valquíria Abrão Coronado Dorce, Osmar Malaspina, Mario Sergio Palma

**Affiliations:** † Scientific and Technological Bioresource Nucleus (BIOREN-UFRO), 28057 Universidad de La Frontera (UFRO), Temuco; Zip Code 4811230; Chile; ‡ Department of Basic and Applied Biology, Institute of Biosciences of Rio Claro, São Paulo State University (UNESP), Rio Claro, SP Zip Code 13506-900; Brazil; § Laboratory of Biological Research, 67688 Amazon College/Amazon University (UNAMA), Santarém, PA 68010-200, Brazil; ∥ 196591 Butantan Institute, São Paulo, SP 05503-900, Brazil

**Keywords:** Toxin library, mass spectrometry, scorpion venom, metabolomics, venomics, exometabolome, LC-MS, ESI-IT-TOF/MS, *Tityus* species

## Abstract

Scorpion venoms consist of proteins, peptides, and various low molecular weight (LMW) organic compounds, which act as toxins. Despite their potential significance, these compounds in scorpion venoms have been little investigated and their full range has not been well characterized. In this work, a targeted metabolomic approach was used in combination with an HPLC-QTOF-MS methodology to create a library of 55 LMW standard compounds, for the analysis of venoms from three *Tityus* species scorpions. This strategy enabled reliable identification of 45 compounds, including 20 amino acids, 4 organic acids, 12 biogenic amines, 6 nitrogenated bases and derivatives, 2 β-carboline-derived alkaloids, and 1 amphetamine. Most of the compounds identified were neurotransmitters and/or neurotoxins, while others can act as homeostasis disruptors or affect the diffusion of venom through the bodies of victims. Therefore, the LMW organic compounds in scorpion venoms play roles in the killing or paralyzing of prey, as well as in defense against large predators.

## Graphical abstract

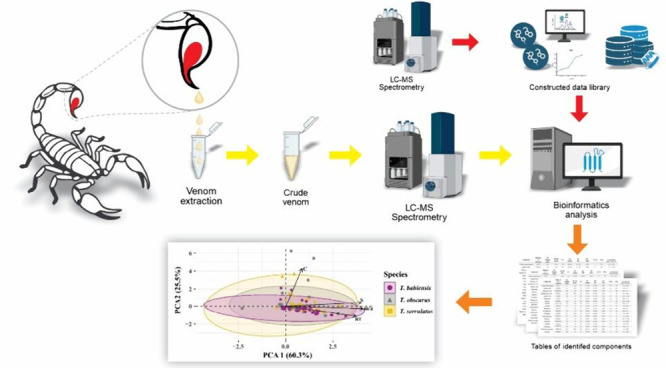

## Introduction

Recent advances in omics technologies have greatly improved understanding of venom components.^1^ These methodologies have revealed that venomous animals are an abundant source of diverse bioactive compounds including proteins, peptides, and many nonpeptidic organic toxins. The compounds in the last class (lipids, free amino acids, and organic acids), collectively known as low molecular weight (LMW) organic compounds,^2,3^ perform the general function of toxins. However, despite the pharmacological importance of these substances, sophisticated analytical platforms have been underutilized in venom studies. Consequently, the literature on animal venoms reports only a limited range of LMW compounds,^4,5^ revealing the continuing need to identify and characterize these components.

The metabolites produced by the venom gland tissue are secreted into the lumen of the gland, so they can be considered as extracellular components. In venomous arthropods, LMW organic toxins may also be biosynthesized by other glands and secreted into the hemolymph, followed by transport to the venom glands, where they are sequestered and stored.^6^ These compounds are exometabolites that act in other organisms, after being injected into the victims of scorpion stinging.^7,8^

The metabolome is the qualitative/quantitative complement of LMW metabolites of an organism or tissue, under specific physiological conditions.^9^ If the complement of LMW toxins from animal venom is composed of extracellular metabolites, the chemical profile of these compounds can be considered a venom exometabolome.

Metabolomic studies of animal venom have adopted two different experimental strategies, namely targeted and untargeted approaches. Venom metabolic profiling has employed a targeted approach, focusing on molecules already identified as toxins, or those with potential pharmaceutical applications. However, few studies have undertaken holistic quantitative analyses of venom metabolite composition across various species, the parameters that influence it, or the similarities and differences in venom metabolic profiles among species.

In studies using targeted metabolomics, snake venoms have been investigated in the search for steroids^10^ and polyamines.^11,12^ This strategy has also been used to investigate steroids in toad venoms.^13,14^ However, most of the research concerning animal venom metabolomics has used untargeted strategies. Examples are investigations of the presence of organic acids, nucleosides, amines, and polyamines in spider venoms,^15,16^ amino acids and amines in scorpion venoms,^17^ amino acids, bufadienolides, and alkaloids in toad venoms,^10^ kynurenic acid, amino acids, and sugars in frogs,^18^ and amino acids, amines, and nucleosides in solitary and social wasps.^19,20^

Annually, over 1.5 million scorpion sting cases are reported worldwide, with outcomes ranging from mild local reactions to severe health risks or death.^21^ In Brazil, the *Tityus* genus is responsible for the most clinically significant scorpion envenomation, with *Tityus serrulatus*, *T*. *bahiensis*, and *T*. *obscurus* being responsible for the majority of human scorpion accidents.^22^ *Tityus serrulatus* is the most dangerous scorpion species in Brazil, causing the most severe envenomation. It is widely distributed across several states, including São Paulo, Minas Gerais, and Rio de Janeiro.^23^ *T*. *bahiensis* causes the most accidents in the Southeast, South, Midwest, and Bahia regions. In the Brazilian Amazon, *T*. *obscurus* is a primary species of medical concern.^24^ In most cases, envenomation typically presents with local symptoms such as pain, swelling, and heat, as well as more general outcomes including headaches and sudoresis. Also common are digestive problems such as vomiting, neurological effects including tremors and dizziness, cardiovascular symptoms such as irregular heartbeats, and breathing difficulties.^8^ The recommended treatment for scorpion stings is the administration of scorpion antivenom, derived from horses hyperimmunized with *T*. *serrulatus* venom. Symptomatic treatment focuses on pain relief, using either injection of 2% lidocaine, without a vasoconstrictor, at the sting site, or oral or parenteral administration of dipyrone or other analgesics.^3^

The search for bioactive molecules in animal venoms can contribute to the discovery of new candidates for pharmacological and industrial applications, in addition to providing a theoretical and technical basis for treating scorpionism. To improve these aspects, it is essential to understand the intra- and interspecific variability of scorpion venoms, as well as the influence of species distribution on their compositions.

The present work explores the metabolomic complexity of these venoms, identifying the metabolites associated with specific effects of scorpion sting envenomation. Mass spectrometry-based targeted metabolomics was used to identify and categorize metabolites in the venoms of three Brazilian *Tityus* species. Chromatographic separation of standard compounds, coupled with high-resolution mass spectrometry analysis, enabled the identification and quantification of metabolites.

## Experimental Section

### Chemical Standards

The chemical standards used were as follows: 1,3-diaminopropane, 2-phenylethylamine, aspartic acid, glutamic acid, 3,4-dihydroxyphenylacetic acid, 4-hydroxyphenylacetic acid, 5-hydroxyindoleacetic acid, indoleacetic acid, malic acid, adenosine diphosphate, adenine, 3–5-cyclic adenosine monophosphate, adenosine 5-monophosphate, adenosine, alanine, arginine, asparagine, betaine, cadaverine, cytosine, dopamine, epinephrine, spermidine, spermine, phenylalanine, gamma-aminobutyric acid (GABA), glycine, glutamine, guanosine, guanine, guanosine monophosphate, guanosine, hydroxytrypargine, histamine, histidine, isoleucine, kainic acid, leucine, lysine, methionine, octopamine, proline, putrescine, serine, taurine, thymine, trypargine, tyramine, tyrosine, threonine, tryptophan, uracil, uridine, and valine. These compounds were obtained from Sigma-Aldrich (St. Louis, MO, USA).

### Biological Materials

Three common Brazilian scorpion species were chosen for this study: *T*. *serrulatus*,^25^ *T*. *bahiensis*,^26^ and *T*. *obscurus* ^27^ (all Scorpiones: Buthidae). *T*. *serrulatus* and *T*. *bahiensis* were reared and maintained at Butantan Institute (São Paulo, Brazil), and were supplied for the present study by the Department of Pharmacology of Butantan Institute. *T*. *obscurus* scorpions were collected in the region of Santarém (Amazonas state, in northern Brazil) by staff of Butantan Institute, with SISBIO/IBAMA authorization (protocol numbers 21483–2 and 20158–1). The animals were maintained in plastic boxes, with water provided *ad libitum* and regular feeding with cockroaches. Access to the genetic patrimony of this scorpion species was formally authorized by CGEN (protocol 010803/2013–0).

### Venom Extraction

The venoms were obtained by electric stimulation of the telsons and collection using micropipettes, followed by lyophilization and storage at – 80 °C. Before analysis, the venom was solubilized in 50% (v/v) acetonitrile, filtered through an Amicon 3000 filter (Millipore), and centrifuged at 8,000*g* for 15 min at 4 °C. The pellets were discarded and the supernatants were lyophilized and stored at – 80 °C until further use. Three batches of crude venom from each species were processed to prepare three replicates.

### Chromatographic and Mass Spectrometric Analysis for Library Construction

Construction of the compound library began with optimization of the conditions for chromatographic separation, using an ultrafast liquid chromatograph (UFLC, Shimadzu) equipped with two LC-20AD pumps and an SIL-20AHT autosampler (Shimadzu). The analytes were separated on an XBridge BEH130 C18 column (2.1 × 100 mm, 3.5 μm) maintained at 38 °C. The mobile phases were (A) ultrapure water with 0.1% (v/v) heptafluorobutyric acid (HFBA), and (B) acetonitrile with 0.1% (v/v) HFBA. The gradient elution started at 2% (v/v) B and increased to 90% (v/v) B over 90 min. The eluent flow rate was 0.2 μL/min and the injection volume was 2 μL.

Mass spectrometric analysis employed a semi-μLC-ESI-microOToF-Q III instrument (Bruker Daltonics) coupled to the UFLC. The mass spectrometer was calibrated (in positive mode) using a 10 mM sodium formate solution, with calibration errors kept within 5 ppm and a calibration score of at least 90%. DataAnalysis v4.1 software (Bruker Daltonics) was used for data acquisition and processing.

The mass spectrometer was operated in both MS and MS/MS modes, with a scan range of *m*/*z* 50–650 and an acquisition rate of 1 Hz. The source parameters were set as follows: voltage of 4500 V, nebulizer gas pressure of 3 bar, drying gas flow rate of 8 L/min, drying gas temperature of 200 °C, and prepulse time of 4 μs. The collision energy was set at 8 eV for the MS mode and at 15 eV for the MS/MS mode. Broadband collision-induced dissociation (bbCID) was used to enable the simultaneous acquisition of MS and MS/MS data.

### Quantification Methodology

The quantification employed QuantAnalysis v2.1 software (Bruker Daltonics), which allowed dynamic analysis of data from the total ion chromatogram (TIC) and facilitated the construction of calibration curves for the compounds. LC-MS/MS data were collected for each standard at four concentrations (3, 15, 69, and 125 ng/μL), with each concentration analyzed in triplicate. This approach enabled the generation of precise calibration curves for all the compounds, with calculation of the line equations and linear regression coefficients (R). Data management and further analyses were performed using Microsoft Excel (version 14).

### Analysis of LMW Compounds in Scorpion Venoms

The software packages used to construct the library and analyze data for the standard compounds and the scorpion venoms were Compass, QuantAnalysis v2.1, and DataAnalysis v4.1 (Bruker Daltonics). Compass was used for chromatographic and mass spectrometric data acquisition. DataAnalysis v4.1 was used to visualize and process the LC-MS data. This software was also used to determine the values of the parameters used to construct the library, including the retention times of the analytes, the *m*/*z* values of the protonated molecules of the compounds, the chromatographic peak areas, and the most important fragment ions generated for each compound in MS/MS mode. Additionally, high-resolution mass spectrometry data were used to determine the molecular formula of each compound, for both the library compounds and the venom samples.

After constructing a library of standard compounds, TargetAnalysis v1.2 software (Bruker Daltonics) was used for chromatographic fractionation, identification, and quantification of the compounds in the scorpion venoms. These analyses were performed using 30 μg of the venom extract from each scorpion species. The identification and validation of the compounds employed the exact *m*/*z* ratios (from high-resolution MS analysis), retention times, presence of qualifying ions (fragment ions), and isotopic patterns. TargetAnalysis software was used to determine the exact mass of each compound in the library, as a quasi-molecular [M + H]^+^ ion. This application also allowed the determination of mSigma, calculated based on the correlation between the theoretical monoisotopic pattern and that obtained experimentally for the ion of interest, providing additional validation by means of a score for the presence of the target compound. The deviations between the values for the validation parameters, such as retention time and *m*/*z*, as well as the presence of qualifying ions, provided a reliability score for the presence of the target compound. After completing the searches using TargetAnalysis, the results were exported to Microsoft Excel v14 (Microsoft, 2010) and organized in tables. The results obtained were manually checked by verifying the mass spectrometry and chromatography data using DataAnalysis. Subsequently, the concentration of each compound was calculated and expressed as μg of compound/μg of venom.

For the compound searches and identification using TargetAnalysis, the parameters were set as follows: 30 s retention time variation, 20 mDa tolerance between theoretical and expected *m*/*z* values for molecules in the monoprotonated form, signal-to-noise ratio limit of 5:1, narrow range mass tolerance value of 10 mDa, wide range mass tolerance of 20 mDa, narrow range mSigma value of 50, and wide range mSigma value of 1000. The initial construction of the metabolite library was performed considering a retention time interval limit among the points of the calibration curve (0.5 min), standardization of the peak area ratio with the concentration of each compound, and determination of the concentrations required to obtain sufficiently high signal intensities to construct reliable curves. The data set that made up the metabolite library was transferred to an Excel spreadsheet for storage in. csv format, including the following parameters: compound name, *m*/*z* of the molecule in the monoprotonated form, retention time, molecular formula, and main fragment ions.

Data processing with the TargetAnalysis software enabled wide- and narrow-range threshold values to be established for the analytical parameters mentioned above. These values were used to determine the detection score for each analyte. The analytical parameters outlined above were used to score and classify the identification of each analyte in the scorpion venoms.

### Data Analysis

Pearson correlation analysis was used to elucidate the behaviors of the concentrations of the identified compounds, considering the three *Tityus* species and the data for the retention times, *m*/*z* values, and compound scores. A correlation matrix was constructed, with the coefficients obtained using the {rcorr} function in the “Hmisc” package^28^ of RStudio v4.3.2 statistical software.^29^ Subsequently, the data were fitted to a Gaussian model to identify any significant relationships between the variables. To assess the dispersion of the compounds isolated from the venoms of the *Tityus* species, principal component analysis (PCA) was performed using the “ade4” package.^30,31^ The “factominer”^32^ and “factoextra”^33^ packages were used to indicate explanatory and quantitative variables, respectively. Ellipses were plotted to show the separation of the different species for the identified compounds (95% confidence level). Species scores, representing the degree of correlation between the mean variables and the principal components, were added to represent each compound identified, for the distribution along the first two principal component axes. The graphical representation was obtained using the “ggplot2” package.^34^

## Results and Discussion

The low molecular weight (LMW) standard compounds were separated using reversed-phase ion-pair chromatography (RPIP-HPLC), with ion-pair formation facilitated by the addition of heptafluorobutyric acid (HFBA) to both mobile phases (A and B). This modification significantly improved the retention of the analytes on the C18 column, consequently enhancing the chromatographic resolution. The formation of ion-pair compounds with HFBA under reversed-phase conditions enabled satisfactory chromatographic separation of the analytes, especially considering the compositional complexity of scorpion venom as an analytical matrix.

Fifty-five LMW standards were analyzed using RPIP-HPLC coupled with an ESI-microTOF-QIII mass spectrometer. Table 1 provides comprehensive analytical data, including the high-resolution molecular masses of quasi-molecular ions, retention times, chemical formulas, compound names, and diagnostic fragment ions (*m*/*z*1, *m*/*z*2, and *m*/*z*3).

**Table 1:** Standard Compounds Used to Build the Library of Low Molecular Mass Compounds, with Their Respective m/z Values as [M + H]^+^ Ions, Their Retention Time (Rt), Molecular Formula Obtained through HRMS, and Their Main Fragment-Ions Used in Their Identification (**m/z 1, m/z 2**, and **m/z 3**)

					Main fragment-ions		
No.	*m*/*z* (M + H)^+^	Rt (min)	Molecular Formula	Standard compounds	*m*/*z* 1	*m*/*z* 2	*m*/*z* 3
**1**	75.0843	12.00	C3H10N2	1.3-Diaminopropane	58.06		
**2**	122.0891	21.00	C8H11N	2-Phenylethylamine	105.06	91.05	
**3**	134.0448	2.10	C4H7NO4	Aspartic acid	116.03	117.03	
**4**	148.0604	2.80	C5H9NO4	Glutamic acid	130.05	102.05	84.04
**5**	169.0422	9.00	C8H8O4	3,4-Dihydroxyphenyl acetic acid	123.04		
**6**	152.0473	6.00	C8H8O3	4-Hydroxyphenyl acetic acid	135.03	152.05	
**7**	192.0582	11.50	C10H9NO3	5-Hydroxyindoleacetic acid	146.06	174.06	
**8**	176.0633	23.00	C10H9NO2	Indoleacetic acid	130.06	158.87	
**9**	214.1001	14.50	C10H15NO4	Kainic acid	196.09	168.10	150.09
**10**	117.0109	1.50	C4H4O4	Maleic acid	99.01	134.04	
**11**	428.0294	1.50	C10H15N5O10P2	Adenosine diphosphate	348.07	136.06	
**12**	136.0618	7.30	C5H5N5	Adenine	119.04		
**13**	330.0525	3.00	C10H12N5O6P	3′,5′-cyclic adenosine monophosphate	136.06	177.06	
**14**	348.0704	1.50	C10H14N5O7P	Adenosine monophosphate	250.09		
**15**	268.0967	3.60	C10H13N5O4	Adenosine	136.06	136.06	
**16**	90.0550	3.00	C3H7NO2	Alanine			
**17**	175.1790	10.00	C6H14N4O2	Arginine	158.09	129.11	116.07
**18**	133.0608	2.00	C4H8N2O3	Asparagine	116.03	88.04	
**19**	118.0784	2.00	C5H10NO2	Betaine	235.16		
**20**	103.1230	13.20	C5H14N2	Cadaverine	86.09		
**21**	112.0432	4.50	C4H5N3O	Cytosine	95.02		
**22**	154.0789	10.50	C8H11NO2	Dopamine	137.06	123.04	
**23**	184.0895	7.00	C9H13NO3	Epinephrine	166.09	135.08	
**24**	146.1652	22.00	C7H19N3	Spermidine	129.14		
**25**	203.2230	27.60	C10H26N4	Spermine	129.14		
**26**	166.0859	17.60	C9H11NO2	Phenylalanine	120.08	149.06	
**27**	104.0706	4.50	C4H9NO2	GABA	87.04	58.07	
**28**	76.0393	2.00	C2H5NO2	Glycine			
**29**	147.0764	2.30	C5H10N2O3	Glutamine	130.05	101.07	84.04
**30**	152.0567	6.00	C5H5N5O	Guanine	135.03		
**31**	284.0989	7.00	C10H13N5O5	Guanosine	152.06	135.03	
**32**	364.0653	1.50	C10H14N5O8P	Guanosine monophosphate	152.06		
**33**	444.0243	1.50	C10H15N5O11P2	Guanosine diphosphate	152.06		
**34**	288.1746	23.50	C15H21N5O	Hydroxytrypargine			
**35**	112.0869	13.50	C5H9N3	Histamine	95.06		
**36**	156.0694	7.00	C6H9N3O2	Histidine	139.06	95.06	81.05
**37**	132.0655	1.80	C5H9NO3	Hydroxyproline	86.06		
**38**	132.1019	14.00	C6H13NO2	Isoleucine	102.06	86.10	
**39**	132.1019	14.50	C6H13NO2	Leucine	86.09	89.00	
**40**	147.1055	8.50	C6H14N2O2	Lysine	130.08	84.09	
**41**	150.0510	10.50	C5H11NO2S	Methionine	102.06	133.03	104.05
**42**	154.0863	7.00	C8H11NO2	Octopamine	136.07	119.04	
**43**	163.1156	16.00	C10H14N2	Nicotine	132.08	120.09	106.07
**44**	116.0706	3.00	C5H9NO2	Proline			
**45**	89.1073	12.00	C4H12N2	Putrescine	72.08		
**46**	106.0499	2.00	C3H7NO3	Serine	88.04	60.04	
**47**	177.0949	15.00	C10H12N2O	Serotonin	160.07	159.09	146.06
**48**	126.0219	1.20	C2H7NO3S	Taurine			
**49**	127.0429	3.00	C5H6N2O2	Thymine	110.10		
**50**	138.0840	13.00	C8H11NO	Tyramine	121.06	107.05	
**51**	182.0738	13.00	C9H11NO3	Tyrosine	165.06	164.07	136.08
**52**	120.0582	13.00	C4H9NO3	Threonine	103.04	85.04	
**53**	272.1796	31.00	C15H21N5	Trypargine	213.14	255.16	196.11
**54**	205.0898	21.50	C11H12N2O2	Tryptophan	188.07	159.09	
**55**	113.0346	1.50	C4H4N2O2	Uracil	113.03	95.01	

The standard compounds were quantified using a standardized calibration routine, as shown in the Supporting Information (Supplementary Figures S1–S16). The calibration curves exhibited excellent linearity, with R-values approaching 1.000, confirming the reliability and reproducibility of the method.

Following method standardization, venom samples from the three *Tityus* species (*T*. *serrulatus*, *T*. *bahiensis*, and *T*. *obscurus*) were fractionated and analyzed under optimized conditions. The total ion chromatograms (TICs) for each species are shown in Figure 1.

**Figure 1:**
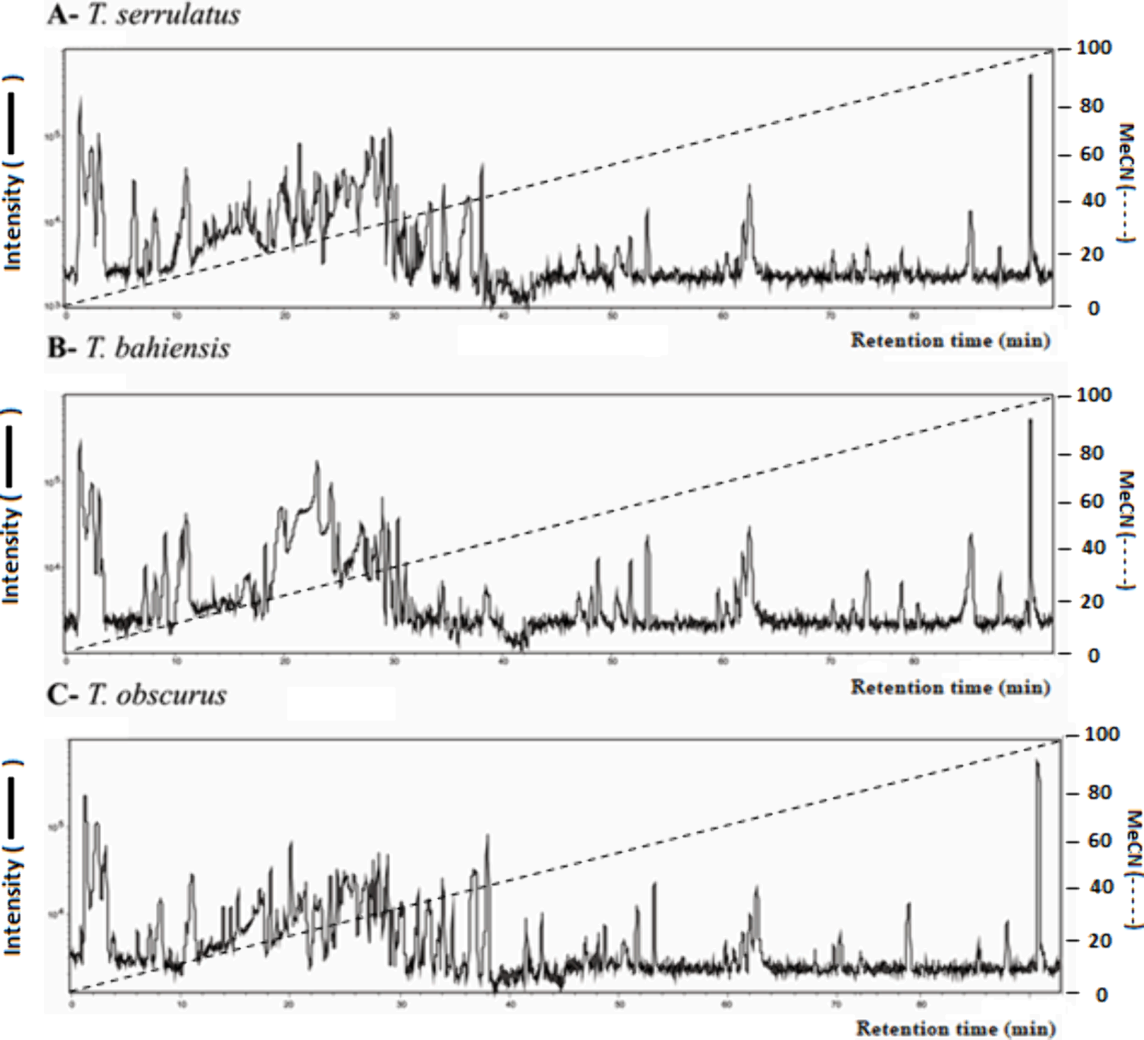
Total Ion Chromatogram (TIC) of the extracts containing the low molecular mass compounds from scorpion venoms, under RP-LCMS using a XBridge BEH130 C18 column (2,1 × 100 mm; 3,5 μm), with a gradient from the mobile phases (A) [H_2_O containing 0.1% (v/v) HFBA] to (B) [MeCN containing 0.1% (v/v) HFBA] in 90 min, at a flow rate of 0.2 mL/min under at 38 °C. The TICs above correspond to (A) *T*. *serrulatus*, (B) *T*. *bahiensis*, and (C) *T*. *obscurus*.

Tables 2–4 show the analytical parameters, including the retention times for the venom samples and standards, mass errors, identification scores, peak areas, and estimated concentrations. These data provide a comprehensive overview of the LMW compound compositions of the venom samples.

**Table 2:** Main Low Molecular Weight Compounds Identified in the Venom of *Tityus serrulatus* by LC-ESI-microTOF-QIII [WATERS XBridgeBEH130 C 18 column (2.1 × 10 mm, 3.5 μm)] from the Comparison with the Metabolite Library Using the Standard Chromatographic Conditions [Containing HFBA 0.1% (v/v)]^a^

*Compound*	*Molecular Formula*	*Rt exp*. *(min)*	*Rt std*. *(min)*	*dRt*	*m/z sample*	*m/z std*.	*Err (Da)*	*Score*	*Area*	Concentration *(ppm ± Std*. *dev*.*)*
Aspartic acid	C8H8O4	2.09	2.10	–0.01	134.0501	134.0448	0.0053	++++	235	1.91 ± 0.40
Kainic acid	C10H9NO3	14.67	14.50	0.17	214.1232	214.1001	0.0231	++	5873	11.62 ± 0.23
Glutamic acid	C10H15NO4	2.06	2.80	–0.74	148.0701	148.0604	0.0097	++++	2117	23.45 ± 0.47
Indoleacetic acid	C4H7NO4	23.06	23.00	0.06	176.0910	176.0633	0.0277	++	13433	103.71 ± 2.60
Adenine	C10H15N5O10P2	7.54	7.30	0.24	136.0599	136.0618	–0.0019	+++	283	0.38 ± 0.01
Alanine	C3H7NO2	2.93	3.00	–0.07	90.0710	90.0550	0.016	+++	835	230.00 ± 4.60
Arginine	C6H14N4O2	10.73	10.00	0.73	175.1802	175.1790	0.0012	+++	141137	461.00 ± 9.23
Betaine	C5H10NO2	2.03	2.00	0.03	118.0800	118.0784	0.0016	++++	7823	9.32 ± 0.19
Cadaverine	C5H14N2	12.94	13.20	–0.26	103.1245	103.1230	0.0015	++++	150	3.45 ± 0.07
Cytosine	C4H5N3O	4.65	4.50	0.15	112.0500	112.0432	0.0068	++++	2349	9.57 ± 0.19
Dopamine	C8H11NO2	10.52	10.50	0.02	154.0732	154.0789	–0.0057	+++	1771	29.67 ± 0.59
Epinephrine	C9H13NO3	7.10	7.00	0.10	184.1000	184.0895	0.0105	+++	437	35.12 ± 0.70
Spermidine	C7H19N3	22.16	22.00	0.16	146.1500	146.1652	–0.0152	++++	6675	15.46 ± 0.31
Spermine	C10H26N4	27.80	27.60	0.20	203.2245	203.2230	0.0015	+++	407	1.22 ± 0.02
Phenylalanine	C9H11NO2	17.70	17.60	0.10	166.0800	166.0859	–0.0059	++++	2105	3.78 ± 0.08
GABA	C4H9NO2	4.54	4.50	0.04	104.0901	104.0706	0.0195	+++	466	3.50 ± 0.07
Glycine	C2H5NO2	2.34	2.00	0.34	76.0396	76.0393	0.0003	+++	474	843.00 ± 22.01
Guanosine	C10H13N5O5	7.52	7.40	0.12	284.0900	284.0980	0.0080	+++	393	2.90 ± 0.05
Hydroxytrypargine	C15H21N5O	23.61	23.50	0.11	288.1700	288.1746	–0.0046	++++	2882	11.06 ± 0.23
Histidine	C6H9N3O2	7.33	7.35	0.33	156.0828	156.0694	0.0134	+++	19403	31.62 ± 0.63
Isoleucine	C6H13NO2	14.01	14.00	0.01	132.0998	132.1019	–0.0021	++++	15343	31.20 ± 0.62
Leucine	C6H13NO2	14.34	14.50	–0.51	132.1043	132.1019	0.0024	++++	75982	217.27 ± 4.36
Lysine	C6H14N2O2	8.48	8.50	–0.02	147.1103	147.1055	0.0048	++++	315662	761.00 ± 15.30
Methionine	C5H11NO2S	10.68	10.50	0.18	150.0654	150.0510	0.0144	+++	5679	16.46 ± 0.31
Octopamine	C8H11NO2	7.24	7.20	–0.04	154.0813	154.0863	–0.005	++++	1930	14.71 ± 0.29
Proline	C5H9NO2	3.18	3.00	0.18	116.0810	116.0706	0.0104	+++	5855	20.75 ± 0.43
Tyramine	C8H11NO	12.97	13.00	–0.03	138.0740	138.0840	–0.01	++	8612	48.80 ± 0.98
Threonine	C4H9NO3	13.02	13.15	0.03	120.0700	120.0582	0.0118	+++	919	46.24 ± 0.93
Tryptophan	C11H12N2O2	21.90	21.50	0.40	205.1000	205.0898	0.0102	+++	14865	46.84 ± 0.91
Valine	C5H11NO2	8.76	8.75	0.01	118.0800	118.0863	–0.0063	++++	5065	42.40 ± 0.85

a ***Rt***: retention time (min); ***Rt exp***.: experimentally measured retention time (min); ***Rt std***.: retention time of standard compounds (min); ***dRt***: variation in retention time (min); **m/z sample**: m/z value measured in venom sample; ***m/z std***.: m/z value of the standard compound; ***Err (Da)***: variation in theoretical mass; ***Score***: score of compound identification; **Area**: average of the measured peak areas (arbitrary unit); ***Concentration***: concentration expressed in part per million (***ppm***) **±** standard deviation (***Std***. ***dev***.).

**3:** Main Low Molecular Weight Compounds Identified in the Venom of *Tityus bahiensis* by LC-ESI-microTOF-QIII [WATERS XBridgeBEH130 C 18 column (2.1 × 10 mm, 3.5 μm)] from the Comparison with the Metabolite Library Using the Standard Chromatographic Conditions [Containing HFBA 0.1% (v/v)]^a^

*Compound*	*Molecular Formula*	*Rt exp*. *(min)*	*Rt std*. *(min)*	*dRt*	*m/z sample*	*m/z std*.	*Err (Da)*	*Score*	*Area*	Concentration *(ppm ± Std*. *dev*.*)*
2-Phenylethylamine	C8H11N	21.11	21.00	0.11	122.0904	122.0891	0.0013	++++	360	2.35 ± 0.04
Kainic acid	C10H9NO3	14.67	14.50	0.17	214.1010	214.1001	0.0009	++++	6761	13.38 ± 0.25
Glutamic acid	C10H15NO4	2.60	2.80	–0.2	148.0611	148.0604	0.0007	++++	1222	13.53 ± 0.21
Indoleacetic acid	C4H7NO4	23.08	23.00	0.08	176.0711	176.0633	0.0078	+++	124	0.95 ± 0.02
Maleic acid	C4H4O4	1.46	1.50	–0.04	117.0134	117.0109	0.0025	++++	359	264.56 ± 5.34
Adenosine	C5H5N5	3.75	3.60	0.15	268.0971	268.0967	0.0004	++++	2543	29.62 ± 0.56
Alanine	C3H7NO2	2.93	3.00	–0.07	90.0620	90.0550	0.0070	+++	494	136.36 ± 2.86
Arginine	C6H14N4O2	9.98	10.00	–0.02	175.1801	175.1790	0.0011	++++	6939	22.68 ± 0.38
Cytosine	C4H5N3O	4.61	4.50	0.11	112.0500	112.0432	0.0068	+++	843	3.43 ± 0.05
Dopamine	C8H11NO2	10.72	10.50	0.22	154.0801	154.0789	0.0002	++++	886	14.84 ± 0.24
Epinephrine	C9H13NO3	7.28	7.00	0.28	184.0923	184.0895	0.0028	++++	362	29.09 ± 0.25
Spermine	C10H26N4	27.81	27.60	0.21	203.2241	203.2230	0.0011	++++	401	1.20 ± 0.02
Phenylalanine	C9H11NO2	17.49	17.60	–0.11	166.0899	166.0859	0.0040	++++	764	1.37 ± 0.02
Glycine	C2H5NO2	2.34	2.00	0.34	76.0382	76.0393	–0.0011	+++	169	300.57 ± 5.78
Glutamine	C5H10N2O3	2.31	2.30	0.01	147.0806	147.0764	0.0042	++++	341	3.16 ± 0.06
Guanine	C5H5N5O	6.19	6.00	0.19	152.0569	152.0567	0.2	++++	659	2.34 ± 0.05
Guanosine	C10H13N5O 5	6.93	7.00	–0.07	284.0945	284.0989	–0.0044	+++	1619	11.81 ± 0.18
Hydroxytrypargine	C15H21N5O	23.52	23.50	0.02	288.1711	288.1746	–0.0035	+++	445	1.70 ± 0.03
Histidine	C5H9NO3	6.89	7.00	–0.11	156.0728	156.0694	0.0034	++++	440	0.71 ± 0.01
Isoleucine	C6H13NO2	14.27	14.00	0.27	132.1044	132.1019	0.0025	++++	9594	19.51 ± 0.35
Leucine	C6H13NO2	14.58	14.50	0.08	132.0998	132.1019	–0.0021	+++	62903	179.86 ± 4.01
Lysine	C6H14N2O2	8.56	8.50	0.06	147.1096	147.1055	0.0041	++++	7229	17.42 ± 0.28
Methionine	C5H11NO2S	10.68	10.35	0.33	150.0583	150.0510	0.0073	+++	1223	3.54 ± 0.05
Proline	C5H9NO2	3.18	3.00	0.18	116.0712	116.0706	0.6	++++	180	0.63 ± 0.01
Serotonin	C10H12N2O	15.00	15.00	0.00	177.1101	177.0949	0.0152	+++	159	0.43 ± 0.01
Tyramine	C8H11NO	13.00	13.00	0.00	138.0900	138.0840	0.006	+++	247	1.39 ± 0.02
Tyrosine	C9H11NO3	13.00	13.15	–0.05	182.0803	182.0738	0.0065	+++	39163	158.64 ± 3.45
Threonine	C4H9NO3	13.27	13.15	0.12	120.0601	120.0582	0.0019	++++	596	29.98 ± 0.60
Trypargine	C15H21N5	31.00	31.00	0.00	272.1807	272.1796	0.0011	++++	1796	5.74 ± 0.10
Tryptophan	C11H12N2O 2	21.61	21.50	0.11	205.0957	205.0898	0.0059	+++	2023	6.37 ± 0.13
Valine	C5H11NO2	8.78	8.75	0.03	118.0799	118.0863	–0.0064	+++	736	6.16 ± 0.15

a ***Rt***: retention time (min); ***Rt exp***.: experimentally measured retention time (min); ***Rt std***.: retention time of standard compounds (min); ***dRt***: variation in retention time (min); **m/z sample**: m/z value measured in venom sample; ***m/z std***.: m/z value of the standard compound; ***Err (Da)***: variation in theoretical mass; ***Score***: score of compound identification; **Area**: average of the measured peak areas (arbitrary unit); ***Concentration***: concentration expressed in part per million (***ppm***) **±** standard deviation (***Std***. ***dev***.).

**Table 4:** Main Low Molecular Weight Compounds Identified in the Venom of *Tityus obscurus* by LC-ESI-microTOF-QIII [WATERS XBridgeBEH130 C 18 column (2.1 × 10 mm, 3.5 μm)] from the Comparison with the Metabolite Library Using the Standard Chromatographic Conditions [Containing HFBA 0.1% (v/v)]^a^

*Compound*	*Molecular Formula*	*Rt exp*. *(min)*	*Rt std*. *(min)*	*dRt*	*m/z sample*	*m/z std*.	*Err (Da)*	*Score*	*Area*	Concentration *(ppm ± Std*. *dev*.*)*
1,3-Diaminopropane	C3H10N2	12.31	12.00	0.31	75.0866	75.0843	0.0023	++++	1011	40.98 ± 0.09
2 Phenylethylamine	C8H11N	20.62	21.00	–0.38	122.0900	122.0891	0.9	+++	7305	47.85 ± 0.07
Aspartic acid	C4H7NO4	2.16	2.10	0.06	134.0400	134.0448	–0.0048	+++	492	4.00 ± 0.01
Kainic acid	C10H15NO4	14.61	14.50	0.11	214.1200	214.1001	0.0199	++++	15303	30.30 ± 0.01
Glutamic acid	C5H9NO4	2.78	2.80	–0.02	148.0700	148.0604	0.0096	++++	1412	15.63 ± 0.03
4-Hydroxyphenylacetic acid	C8H8O3	5.79	6.00	–0.21	153.0800	152.0473	1.0327	+++	6201	27.64 ± 0.04
5-Hydroxyindoleacetic acid	C10H9NO3	11.39	11.50	–0.11	192.0900	192.0582	0.0318	+++	8317	58.60 ± 0.12
Indoleacetic acid	C10H9NO2	23.12	23.00	0.12	177.1000	176.0633	1.0367	++++	2923	22.56 ± 0.04
Maleic acid	C4H4O4	1.68	1.50	0.18	117.0670	117.0109	0.0561	++	3358	474.63 ± 7.35
Alanine	C3H7NO2	3.10	3.00	0.1	90.0549	90.0550	–0.1	++++	12682	500.70 ± 9.86
Arginine	C6H14N4O2	10.31	10.00	0.31	175.1811	175.1790	0.0021	++++	19180	62.70 ± 1.36
Asparagine	C4H8N2O3	2.20	2.00	0.2	133.0700	133.0608	0.0092	++	558	13.87 ± 0.02
Cytosine	C4H5N3O	4.48	4.50	–0.02	112.0687	112.0432	0.0255	+++	3379	13.76 ± 0.03
Dopamine	C8H11NO2	10.49	10.50	–0.01	154.0889	154.0789	0.0100	++++	8701	145.77 ± 2.89
Epinephrine	C9H13NO3	6.89	7.00	–0.11	184.1240	184.0895	0.0345	++	150	12.05 ± 0.02
Spermidine	C7H19N3	22.11	22.00	0.11	146.1555	146.1652	–0.0097	++++	5399	12.50 ± 0.01
Spermine	C10H26N4	27.55	27.60	–0.05	203.2218	203.2230	–0.0012	++++	438	1.31 ± 0.01
Phenylalanine	C9H11NO2	17.80	17.60	0.2	166.0880	166.0859	0.0021	++++	10855	19.49 ± 0.38
GABA	C4H9NO2	4.29	4.50	–0.21	104.0934	104.0706	0.0228	++++	632	4.74 ± 0.01
Glycine	C2H5NO2	1.98	2.00	–0.02	76.0401	76.0393	0.8	++++	1249	221.42 ± 4.38
Guanosine	C10H13N5O5	7.15	7.00	0.15	284.0945	284.0989	–0.0044	++++	807	5.88 ± 0.01
Hydroxytrypargine	C15H21N5O	23.19	23.50	–0.31	288.1700	288.1728	–0.0028	++++	33316	127.83 ± 2.27
Histamine	C5H9N3	13.65	13.50	0.15	112.0850	112.0869	–0.0019	++++	3651	10.98 ± 0.02
Histidine	C6H9N3O2	7.13	7.00	0.13	156.0808	156.0694	0.0114	++++	52077	84.87 ± 016
Isoleucine	C6H13NO2	14.21	14.00	0.21	132.0900	132.1019	–0.0119	++++	9054	18.41 ± 0.35
Leucine	C6H13NO2	14.52	14.50	0.02	132.1000	132.1019	–0.0019	++++	7945	22.71 ± 0.03
Lysine	C6H14N2O2	8.55	8.50	0.05	147.1100	147.1055	0.0045	++++	62893	151.63 ± 2.94
Methionine	C5H11NO2S	10.48	10.50	–0.02	150.0600	150.0510	0.0090	++++	11281	32.69 ± 0.06
Octopamine	C8H11NO2	7.03	7.00	0.03	154.0910	154.0863	0.0047	+++	744	5.67 ± 0.01
Proline	C5H9NO2	2.77	3.00	–0.23	116.0900	116.0706	0.0194	++++	1786	6.33 ± 0.11
Putrescine	C4H12N2	12.32	12.00	0.32	89.1000	89.1073	–0.0073	++++	8318	88.32 ± 1.65
Serotonin	C10H12N2O	15.12	15.00	0.12	177.1100	177.0949	0.0151	++++	54698	150.46 ± 2.70
Thymine	C5H6N2O2	2.93	3.20	–0.27	127.0600	127.0429	0.0171	++++	24301	70.73 ± 1.37
Tyramine	C8H11NO	13.03	13.00	0.03	138.0710	138.0840	–0.0130	++	7455	42.23 ± 0.85
Tyrosine	C9H11NO3	13.11	13.00	0.11	182.0800	182.0738	0.0062	++++	4543	18.40 ± 0.34
Threonine	C4H9NO3	12.98	13.15	–0.17	120.0700	120.0582	0.0118	++++	1404	70.64 ± 1.45
Tryptophan	C11H12N2O2	21.45	21.50	–0.05	205.1045	205.0898	0.0147	++++	10460	32.95 ± 0.65
Uridine	C9H13N2O9P	1.56	1.50	0.06	352.0512	352.0431	0.0081	++	18760	167.91 ± 1.42
Valine	C5H11NO2	8.70	8.75	–0.05	118.0800	118.0863	–0.0063	++++	25528	213.71 ± 4.21

a ***Rt***: retention time (min); ***Rt exp***.: experimentally measured retention time (min); ***Rt std***.: retention time of standard compounds (min); ***dRt***: variation in retention time (min); **m/z sample**: m/z value measured in venom sample; ***m/z std***.: m/z value of the standard compound; ***Err (Da)***: variation in theoretical mass; ***Score***: score of compound identification; **Area**: average of the measured peak areas (arbitrary unit); ***Concentration***: concentration expressed in part per million (***ppm***) **±** standard deviation (***Std***. ***dev***.).

To develop the data processing method using the TargetAnalysis software, it was necessary to establish both wide-range and narrow-range threshold values for the parameters mentioned above. These values were used to assign the scores for the detection and identification of the compounds, enabling classification of the parameters. Tables 2–4 show the following analytical parameters for the compounds: experimental retention times for the metabolites in the scorpion venoms, retention times for the standards, differences in retention times between the venom compounds and standards, *m*/*z* of the compounds detected as [M + H]^+^ ions for the venoms and standards, errors of the molecular masses, identification scores, peak areas of the compounds, and concentrations of the compounds detected in the scorpion venoms. All the compounds assigned to the venoms were reliably identified, presenting scores from ++ to ++++.

A library of LMW organic compounds was constructed using 55 standard reference compounds. From this library, 45 compounds were reliably identified in the venoms of the *Tityus* scorpions. Among the compounds identified, 20 were amino acids, 4 were organic acids, 12 were biogenic amines, 6 were nitrogenated bases and/or their derivatives, 2 were alkaloids, and 1 was an amphetamine. The global distribution profile of these compounds across the venoms of the three scorpion species provided insights into the biochemical diversity and specificity of the venom compositions (Table 5).

**Table 5:** Summary of Compounds Detected, Identified and Quantified in the Venom of Each Scorpion Species

	**Quantity in parts per million (ppm)**		
	**Scorpion species**		
**Compounds**	***T***. ***serrulatus***	***T***. ***bahiensis***	***T***. ***obscurus***
1,3-Diaminopropane	-	-	40.98 ± 0.09
2-Phenylethylamine	-	2.35 ± 0.04	47.85 ± 0.07
Adenine	0.38 ± 0.01	-	-
Adenosine	-	29.62 ± 0.56	-
Alanine	230.00 ± 4.60	136.36 ± 2.86	500.70 ± 9.86
Arginine	461.00 ± 9.23	22.68 ± 0.38	62.70 ± 1.36
Asparagine	-	-	13.87 ± 0.02
Aspartic Acid	1.91 ± 0.40	-	4.00 ± 0.01
Betaine	9.32 ± 0.19	-	-
Cadaverine	3.45 ± 0.07	-	-
Cytosine	9.57 ± 0.19	3.43 ± 0.05	13.76 ± 0.03
Dopamine	29.67 ± 0.59	14.84 ± 0.24	145.77 ± 2.89
Epinephrine	35.12 ± 0.70	29.09 ± 0.25	12.05 ± 0.02
GABA	3.50 ± 0.07	-	4.74 ± 0.01
Glutamic acid	23.45 ± 0.47	13.53 ± 0.21	15.63 ± 0.03
Glutamine	-	3.16 ± 0.06	-
Glycine	843.00 ± 22.01	300.57 ± 5.78	221.42 ± 4.38
Guanine	-	2.34 ± 0.05	-
Guanosine	2.90 ± 0.05	11.81 ± 0.18	5.88 ± 0.01
Histamine	-	-	10.98 ± 0.02
Histidine	31.62 ± 0.63	0.71 ± 0.01	84.87 ± 016
5-Hydroxyindoleacetic acid	-	-	58.60 ± 0.12
4-Hydroxyphenylacetic acid	-	-	27.64 ± 0.04
Hydroxytrypargine	11.06 ± 0.23	1.70 ± 0.03	127.83 ± 2.27
Indoleacetic acid	103.71 ± 2.60	0.95 ± 0.02	22.56 ± 0.04
Isoleucine	31.20 ± 0.62	19.51 ± 0.35	18.41 ± 0.35
Kainic acid	11.62 ± 0.23	13.38 ± 0.25	30.30 ± 0.01
Leucine	217.27 ± 4.36	179.86 ± 4.01	22.71 ± 0.03
Lysine	761.00 ± 15.30	17.42 ± 0.28	151.63 ± 2.94
Maleic acid	-	264.56 ± 5.34	474.63 ± 7.35
Methionine	16.46 ± 0.31	3.54 ± 0.05	32.69 ± 0.06
Octopamine	14.71 ± 0.29	-	5.67 ± 0.01
Phenylalanine	3.78 ± 0.08	1.37 ± 0.02	19.49 ± 0.38
Proline	20.75 ± 0.43	0.63 ± 0.01	6.33 ± 0.11
Putrescine	-	-	88.32 ± 1.65
Serotonin	-	0.43 ± 0.01	150.46 ± 2.70
Spermidine	15.46 ± 0.31	-	12.50 ± 0.01
Spermine	1.22 ± 0.02	1.20 ± 0.02	1.31 ± 0.01
Threonine	46.24 ± 0.93	29.98 ± 0.60	70.64 ± 1.45
Thymine	-	-	70.73 ± 1.37
Trypargine	-	5.74 ± 0.10	-
Tryptophan	46.84 ± 0.91	6.37 ± 0.13	32.95 ± 0.65
Tyramine	48.80 ± 0.98	1.39 ± 0.02	42.23 ± 0.85
Tyrosine	-	158.64 ± 3.45	18.40 ± 0.34
Valine	42.40 ± 0.85	6.16 ± 0.15	213.71 ± 4.21

The distribution of LMW toxins among the three *Tityus* species revealed both significant overlap and species-specific profiles. As shown in the Venn diagram (Figure 2), the three species shared 23 compounds, including key amino acids (glutamic, aspartic, and kainic acids), biogenic amines (dopamine and epinephrine), and nitrogenated bases (cytosine and guanosine).

**Figure 2:**
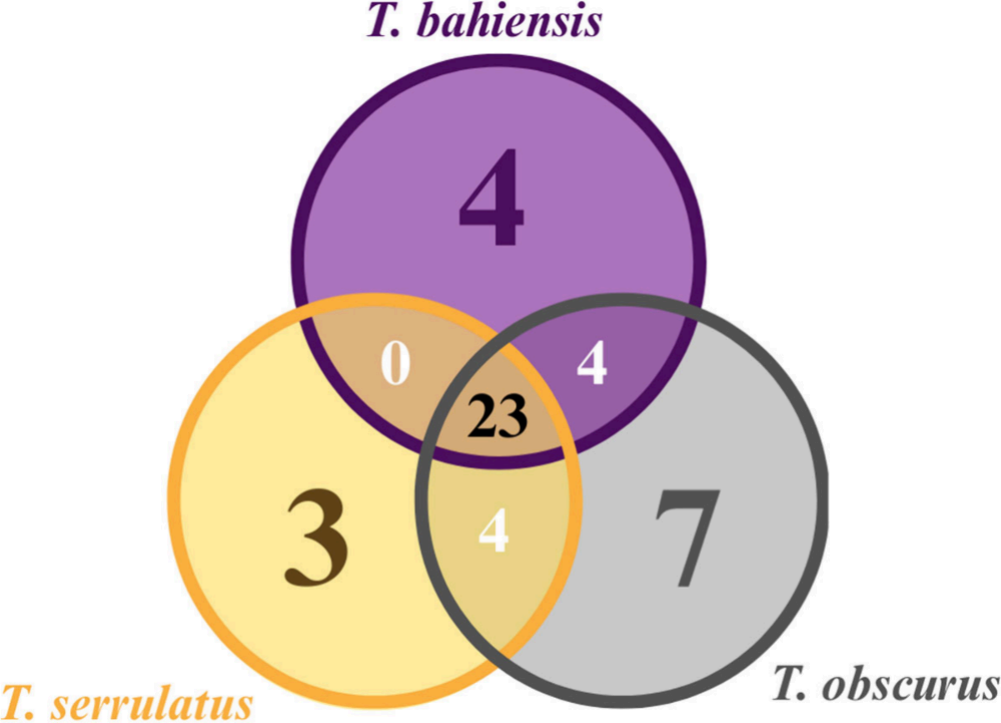
Venn diagram of the low molecular mass compounds detected in the venoms from the scorpions *T*. *serrulatus*, *T*. *bahiensis* and *T*. *obscurus*.

Notably, four compounds were shared between *T*. *serrulatus* and *T*. *obscurus* (GABA, spermidine, octopamine, and aspartic acid). Another four compounds were common to *T*. *bahiensis* and *T*. *obscurus* (tyrosine, maleic acid, serotonin, and 2-phenylethylamine). However, no compound was found to be shared between *T*. *serrulatus* and *T*. *bahiensis*. Unique compounds were identified for each species, with *T*. *serrulatus* presenting betaine, cadaverine, and adenine, while *T*. *bahiensis* presented glutamine, trypargine, adenosine, and guanine, and *T*. *obscurus* presented asparagine, hydroxyphenylacetic acid, hydroxyindoleacetic acid, 1–3 diaminopropane, histamine, putrescine, and thymine.

Several compounds reported previously in other animal venoms, such as taurine, serine, and AMP-related derivatives, were not reliably identified in the *Tityus* venoms, suggesting unique venom profiles and potential evolutionary specialization. These findings highlighted the conserved biochemical elements and distinct molecular signatures of the scorpion species.

The PCA results provided insights into the distribution of venom compounds among the *Tityus* species. The first principal component (PC1), accounting for 60.3% of the variance, was the primary axis determining compound differentiation among the species. The second principal component (PC2) explained 25.5% of the variance, contributing to the structure of the compound distribution (Figure 3). The compound retention time, *m*/*z*, and score were significantly correlated (Gaussian model: df = 3; F = 4.122; p = 0.0078), suggesting that these parameters were important in determining compound variability. However, the compound concentrations showed weak correlations with these parameters, implying that concentration alone did not strongly influence compound classification.

**Figure 3:**
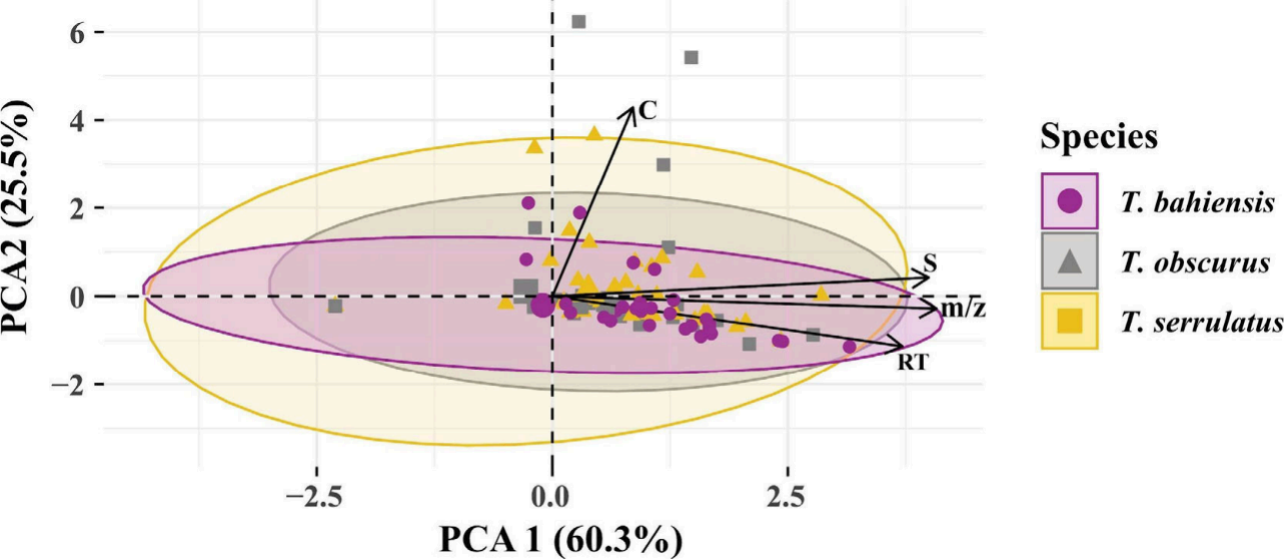
Principal component analysis (PCA) and spatial distribution of proteomics analysis of venom from scorpion species. Plots analyzed *Tityus* species based on the concentrations, retention time, *m*/*z* and score of compounds identified. The species close to each other contain a similar composition of substances, while species with the biggest distance differ in substances identified. The ellipses were described with a 95% confidence level of the sampled venoms, and the arrows show the quantitative parameters identified along the first two principal component axes. **RT**: Retention Time; *m* **/** *z*: mass-to-charge ratio; **S**: Score; and **C**: Concentration.

The species *T*. *bahiensis* and *T*. *serrulatus* showed greater similarity in venom compound distribution, as indicated by the clustering of the points, reflecting shared chemical features of the venom compositions. *T*. *obscurus* appeared to be more distinct, with the corresponding data points distributed differently across the PCA component plot. The larger elliptical spread for *T*. *serrulatus* could be attributed to variations in the compound concentrations, as suggested by the influence of the concentration vector. This variability could have been due to intraspecific differences in venom composition, as well as the effects of environmental and physiological factors on production of the compounds.

The LMW organic compounds found in scorpion venom are metabolites secreted into the lumen of the venom gland, becoming exometabolites that do not act in the classical metabolic steady-state. However, these compounds remain active, playing an important role in the envenomation mechanism, with various pharmacological and physiological actions.

Amino acids found in the venoms of the three scorpion species included glutamic acid, aspartic acid, histidine, arginine, phenylalanine, alanine, glycine, glutamine, isoleucine, leucine, lysine, methionine, asparagine, tyrosine, threonine, tryptophan, proline, and valine. These amino acids are essential components of proteins, although some are also neurotransmitters, such as aspartic acid, glycine, and glutamic acid.^35,36^ In addition to being a proteinogenic amino acid, glutamic acid is the most abundant and important excitatory neurotransmitter in the central nervous system (CNS).^37^ This amino acid was observed in the venoms of the three scorpion species studied in the present work.

Gamma-aminobutyric acid (GABA), which was detected in the venoms of *T*. *serrulatus* and *T*. *obscurus*, is a nonproteinogenic amino acid present at high concentrations in the CNS, where it functions as an inhibitory neurotransmitter and blocks CNS signals. GABA performs a series of functions in neuronal biochemistry and postsynaptic regulation.^38^ A decrease in GABAergic neurotransmission in the CNS contributes to hyperexcitability.^39^ GABA activates the enzymatic processes of transamination and decarboxylation in the Krebs cycle, being synthesized from glutamate by the l-glutamic acid decarboxylase enzyme, with vitamin B6 as cofactor. This process converts the primary excitatory neurotransmitter (glutamate) into one of the main inhibitory neurotransmitters.^39^

Kainic acid was detected in the venoms of all three scorpion species studied. Kainic acid is an agonist of the kainate subtype of ionotropic glutamate receptors, which directly gate ion channels and are generally excitatory. Studies using experimental models have shown that the administration of kainic acid can result in convulsions,^40^ changes in rodent behavior,^41^ oxidative stress,^42^ glial activation,^43^ production of inflammatory mediators,^44^ endoplasmic reticulum stress, mitochondrial dysfunction, and selective neuronal degeneration in the rodent brain.^45^

Betaine (N,N,N-trimethylglycine), present in the *T*. *serrulatus* venom, is an essential intracellular osmolyte that regulates cell volume by counterbalancing changes in extracellular tonicity and stabilizing macromolecules against various physiological disturbances.^46,47^ Betaine also plays a key role in the metabolism of methyl radicals, providing cells with a supply of methyl groups.^46,47^ Betaine can be obtained from the diet or be produced in the body by the oxidation of choline.^48^

The polyamines found in the scorpion venoms included diaminopropane, putrescine, spermidine, cadaverine, and spermine. These compounds are widely distributed in animal tissues, where they play fundamental roles in the action of hormones, control of cell division, and synthesis of macromolecules.^49^ Polyamines have also been reported to influence cellular excitability and neurotransmission by interacting with potassium ion channels and AMPA- and NMDA-dependent glutamate receptors.^11,50^

Serotonin was identified in the *T*. *bahiensis* and *T*. *obscurus* venoms. This compound does not generally appear to alter the toxicity of venoms, but can contribute to the occurrence of pain and inflammation.^51^

Dopamine, which was identified in the venoms of all three scorpion species, is a monoamine neurotransmitter acting in the communication of messages between nerve cells in the brain and the rest of the body, leading to an increased heart rate that could assist rapid circulation of venom components in the body of the prey.^52^

Histamine, identified in the venoms of *T*. *bahiensis* and *T*. *serrulatus*, has been reported previously in other scorpion venoms. This pro-inflammatory compound has a vasodilatory effect on blood vessels, increasing vascular permeability and reducing blood pressure.^53^

Epinephrine, which was observed in the venoms of the three scorpion species, is a hormone that also acts as a CNS neurotransmitter. It participates in the sympathetic nervous system and is involved in “fight-or-flight” responses to emergency situations.^54^

Spermine has been reported in the venoms of spiders^55^ and snakes,^56,57^ while putrescine, spermidine, and cadaverine have been observed in the venoms of four species of tarantulas.^58^ Spermine causes nephrotoxicity,^59^ breathing difficulty, hypotension, and diuresis.^60^ Many of these effects are also exhibited by spermidine, but at higher concentrations of the compound. In mice, spermidine was reported to cause other toxicity symptoms including head and limb tremors, reduced muscle tone, paralysis of the hind limbs, decreased reflexes, hypothermia, and peripheral vasoconstriction,^61^ while in rats, it led to histopathological renal changes.^62^

Octopamine was observed in the venoms of *T*. *serrulatus* and *T*. *obscurus*, while tyramine, an invertebrate neurotransmitter analogous to vertebrate adrenergic transmitters, was detected in the venoms of all three scorpion species. The decarboxylation of tyrosine produces these compounds, with tyramine being the biological precursor of octopamine. Both compounds are neurotransmitters that act by coupling to G-protein receptors. They can be stored at high concentrations in synaptic vesicles, presenting agonist activity on adrenergic receptors in mammals, and can cause sudden hypotension. Octopamine regulates several behaviors and sense organs in insects, enabling appropriate responses to external stimuli. This compound has attracted particular attention, because it is the only biogenic amine acting exclusively in invertebrates, so its receptors are promising targets for new insecticides.^63^ Octopamine and tyramine in animal venoms may cause hyperexcitation in the prey, prior to paralysis.^64^

The venom of *T*. *obscurus* contained 1,3-diaminopropane, which is a compound that acts as a building block in the biosynthesis of acylpolyamine toxins of orb-web spiders.^65^ However, no information is available regarding its physiological and pharmacological effects as a free metabolite in venom.

This is the first study to detect guanosine and cytosine in scorpion venom. Adenosine, which was previously reported in the venom of the scorpion *Heterometrus laoticus*, was reliably identified in the venom of *T*. *bahiensis*. Adenosine is known to be a potent inhibitor of platelet aggregation, consequently interfering in blood coagulation.^66^ Previous work has evidenced the presence of compounds such as guanosine, inosine, and 2,4,6-trihydroxypurine in spider venoms.^67^ Sulfated guanosine derivatives present in brown spider venoms have been found to act as potent neurotoxins.^16^

5-hydroxyindoleacetic and 4-hydroxyphenylacetic acids, which were detected in the *T*. *obscurus* venom, are used in venomous animals as chromophores in the biosynthesis of acylpolyamines.^65^ No pharmacological roles are known for these compounds in animal venoms. Indoleacetic acid was observed in the venoms of the three *Tityus* species. In animal models, the administration of indoleacetic acid was found to cause an acute hypoglycemic response,^68,69^ accompanied by irritability, weakness, myotonia,^68^ lassitude, and immobility.^70^ Maleic acid was found in the venoms of *T*. *bahiensis* and *T*. *obscurus*. This is the first report of maleate in animal venom and it is not known whether this compound is active in scorpion envenomation incidents.

The detected LMW compounds included some beta-carboline alkaloids, such as hydroxytrypargine in the venoms of all three scorpion species and trypargine in the venom of *T*. *bahiensis*. These alkaloid toxins were previously observed in the venoms of the spiders *Nephyla clavipes* and *Parawixia bistriata*,^71,72^ acting as potent neurotoxins causing the death or paralysis of prey.

The amphetamine 2-phenylethylamine was identified in the venoms of *T*. *bahiensis* and *T*. *obscurus*. This compound can induce depression and anxiety, acting on the motor activity of animals, with changes in the frequency parameters of locomotion and body lifting.^73^

Venomous animals typically use their venoms to capture prey, deter predators, facilitate parasitism, and perform extra-oral digestion.^74^ The richness of LMW compounds in animal venom provides many tools for these purposes. It is likely that natural selection has led to the use of these compounds as toxins in animal venoms, due to their capacity to affect neuronal systems. Some of them can induce pain or discomfort in predators (such as vertebrates), targeting monoaminergic systems, apparently for the purpose of defense.^64^ Another potential function of LMW compounds in scorpion venoms is to accelerate the distribution of venom components throughout the body of the victim. For example, histamine and serotonin (which induces the release of histamine) cause vasodilation at the injection site, thereby facilitating the circulation of compounds throughout the victims.^75^ Dopamine has been reported in Hymenoptera venoms, acting to increase the heart rate in the victims of stinging.^52^ Several of the LMW organic compounds found in venoms can activate the sympathetic nervous system or adrenergic receptors in vertebrates, increasing the heart rate.

Another aspect to be considered is the purpose of compromising the ability of the prey to develop an effective escape response. The presence of many neurotransmitters and ion channel blockers may be related to the occurrence of intense physiological stress, which generally results in paralysis and/or death of the prey. Paradoxically, this is often achieved by activation of the sympathetic nervous system, such as in the catecholamine cascade and other actions involving epinephrine, often causing cardiac collapse, as demonstrated in the case of spider venoms.^76^ The hyperexcitation induced by the venom immobilizes the prey until the occurrence of flaccid paralysis, characterized by weakness or reduced muscle tone. Hyperarousal in the prey is a common response to the venoms of various animals, such as spiders, scorpions, coelenterates, and some snails, associated with the presence of monoamines in the venoms.^76–79^ Hyperexcitation results in immediate immobilization of the prey, so that it remains within reach of the predator until flaccid paralysis begins. It has been found that dopamine in the venom of the *Amulex compressa* wasp induces excessive grooming in cockroaches, preventing their escape,^77^ so an analogous mechanism may occur in the case of scorpion venom.

## Conclusions

Generally venomous animals use their venoms as instruments for prey capture, and for deterring predators, facilitating parasitism, and to perform extra-oral digestion. The richness of LMW compounds in animal venoms certainly provide many tools for the above-mentioned purposes. Many of these compounds act individually at physiologic/pharmacological level, while others may interact with the proteins/peptides (specially neurotoxins and their receptors), potentiating their effects.

One of the reasons why natural selection chosen these compounds to act as toxins in animal venoms is probably related to their capacity of affecting neuronal systems, as well because some of them can produce pain or discomfort in predators (e.g., vertebrates), targeting monoaminergic systems, apparently with a defensive purpose. Another potential function for LMW compounds from scorpion venoms is to accelerate the distribution of venom components throughout the victim’s body. Some LMW organic compounds from venoms can activate the proteins of the sympathetic nervous system and the adrenergic receptors, thereby increasing heart rate in vertebrates.

Another point to be taken into consideration is the purpose of compromising the prey’s ability to organize an effective escape response. The presence of a large number of different types of neurotransmitters and ion channel blockers may be related to the occurrence of intense physiological stress, which generally results in paralysis and/or death of prey. Paradoxically, this is often accomplished with activation of the sympathetic nervous system, as in the catecholamine cascade and other actions involving epinephrine, often causing cardiac collapse. The hyperexcitation induced by the venoms immobilizes the prey until the occurrence of flaccid paralysis, which is characterized by weakness or reduced muscle tone. Hyperarousal is a common strategy perceived as responses to various types of animal venoms, produced by monoamines present in these venoms.

Scorpion venoms are extremely versatile tools, being effective against prey (insects) and in defense against vertebrate predators. These venoms have drastic effects in mammals, and an interesting fact to speculate is why the venoms evolved this way, since these animals normally target insects as prey. The action in mammals is possibly related to the effect caused by the synergistic actions at the pharmacological level, of the various biologically active LMW organic compounds present in the venoms, causing considerable physical discomfort in mammals’ victims of stings of these animals, with a powerful defensive effect. In addition to this, the high number of compounds acting at the level of the nervous and homeostatic systems, can cause an intense physiological stress that results in the paralysis or death of scorpions’ prey.

## Supplementary Material



## Notes

Classification of the analytical parameters obtained in the identifications of the venom compounds; scoring criteria for identification of the analytes in scorpion venoms; regression and angular coefficients of the calibration curves for the different compounds in the library; standard compound calibration curves for the analytes identified in scorpion venom (PDF)
