# Supplementary material for: Targeted Metabolomics of Tityus Scorpion Venoms: Unveiling Small-Molecule Components
Source: J Am Soc Mass Spectrom. 2025 Oct 7;36(11):2489–501. doi: 10.1021/jasms.5c00238 (PMC12598862; doi:10.1021/jasms.5c00238)
Supplement: Supplementary file 1 [file js5c00238_si_001.pdf]

Targeted Metabolomics of *Tityus* Scorpion Venoms: Unveiling Small-Molecule Components

Nathalia Baptista Dias[1,*], Bibiana Monson de Souza[2], Geovanny Barroso[2], Javier Ortiz Leiva[1], Gabriela Mendonça Paula[2], Hipócrates M. Chalkidis[3], Valquíria Abrão Coronado Dorce[4], Osmar Malaspina[2], Mario Sergio Palma[2,*]

[1]Scientific and Technological Bioresource Nucleus (BIOREN-UFRO), Universidad de La Frontera (UFRO), Temuco; Zip Code 4811230; Chile.

[2]Department of Basic and Applied Biology, Institute of Biosciences of Rio Claro, São Paulo State University (UNESP), Rio Claro, SP, Zip Code 13506-900; Brazil.

[3]Laboratory of Biological Research, Amazon College/Amazon University (UNAMA), Santarém, PA, Zip Code 68010-200; Brazil.

[4]Butantan Institute, São Paulo, SP, Zip Code 05503-900; Brazil.

---

*Corresponding authors: Dra. Nathalia Baptista Dias, nathalia.dias@ufrontera.cl, +56 45 273419/ Dr. Mario Sergio Palma, mario.palma@unesp.br, +55 19 3526 4163

Supplementary Tables

Table S1. Classification of the analytical parameters obtained in the identifications of the venom compounds based on the threshold values (wide and narrow ranges) of the analytical parameters *.

**Parameter measured**	**Conditions**	**Classification**
Mass accuracy (M)	0 ≤ d*m/z* ≤ MT1	Excellent
	MT1 < d*m/z* ≤ MT2	Good
	MT2 < d*m/z*	Bad
mSigma value (mS)	0 ≤ mS ≤ mST1	Excellent
	mST1 < mS ≤ mST2	Good
	mST2 < d*m/z*	Bad
Retention time (Rt)	0 ≤ dRt ≤ TRT1	Excellent
	RtT1 < dRt ≤ RtT2	Good
	RtT2 < dRt	Bad
Qualifier ions	Identification of at least one qualifier ion with 0 ≤ d*m/z* ≤ tolerance of identification	Excellent
	tolerance of identification < d*m/z*	Bad

(*) **d*m/z***: is the deviation of the experimental m/z value determined for each compound, expressed in mDa; **MT1:** is the narrow range threshold value of mass accuracy tolerance for each compound, expressed in mDa; **MT2**: is the wide range threshold value of mass accuracy tolerance for each compound expressed in mDa; **mS:** is the *mSigma* value, which is the deviation of the theoretical pattern of each compound from the measured isotopic pattern, used as “qualifier factor”; **mST**: is the narrow range threshold value of quality of the tolerance factor of each compound; **mST2**: is the wide range threshold value of quality of the tolerance factor of each compound; **dRt**: is the deviation of the measured retention time from expected one for each compound, expressed in minutes; **RtT1:** is the narrow range threshold value of retention time tolerance for each compound, expressed in minutes; **RtT2:** is the wide range threshold value of retention time tolerance for each compound, expressed in minutes.

Table S2. Criteria of scoring for the identification of the analytes of scorpion venoms based on the quality of the analytical parameters.

**Score classification**	**Interpretation**	**Symbol**
4 excellent parameters	very high reliability of compound identification	++++
3 excellent parameters	high reliability of compound identification	+++
2 excellent parameters and 2 good parameters	reliable identification	++
1 or more bad parameters (independent of the number of excellent or good parameters	Not reliable identification	-

Table S3. Results of the regression and angular coefficients of the calibration curves for the different compounds in the library

Compounds	R^2	Angular coefficient (a)
**1.3-diaminopropane**	0.9973	822232.20
**2-Phenylethylamine**	0.9668	4683414.31
**Indoleacetic acid**	0.9992	4317545.72
**4-Hydroxyphenylacetic acid**	0.9987	7476686.78
**5-Hydroxyindole acetic acid**	0.9999	4730558.57
Aspartic acid	0.9984	4099741.42
**Kainic acid**	0.9991	16834174.30
**Glutamic acid**	0.9975	3009591.88
**Maleic acid**	0.9997	45232.23
**Adenine**	0.9991	24755875.19
**Adenosine**	0.9994	2861324.89
**Alanine**	0.9984	120756.47
**Arginine**	0.9998	10196039.16
**Asparagine**	0.9993	1340524.52
**Betaine**	0.9998	3659253.11
**Cadaverine**	0.9998	1449831.63
**Cytosine**	0.9976	8182604.72
**Dopamine**	0.9990	1989662.61
**Epinephrine**	0.9981	414770.91
**Spermidine**	0.9995	14393524.67
**Spermine**	0.9885	11131481.51
**Phenylalanine**	0.9983	18562930.91
**GABA**	0.9992	4436602.98
**Glycine**	0.9973	18741.74
**Glutamine**	0.9978	3593696.32
**Guanine**	0.9998	9385739.76
**Guanosine**	0.9970	4567735.11
**Hydroxytripargine**	0.9984	8687272.20
**Histamine**	0.9981	11083225.69
**Histidine**	0.9985	20452959.31
**Isoleucine**	0.9982	16387326.50
**Leucine**	0.9997	11657272.49
**Lysine**	0.9921	13825543.75
**Methionine**	0.9983	11500345.69
**Octopamine**	0.9855	4372695.52
**Proline**	0.9898	9403381.00
**Putrescine**	0.9277	3139009.75
**Serotonin**	0.9998	12117367.34
**Thymine**	0.9992	11452731.30
**Tyramine**	0.9977	5883375.68
**Tyrosine**	0.9906	8228519.55
**Threonine**	0.9996	662480.58
**Trypargine**	0.9978	10412243.63
**Tryptophan**	0.9996	10579447.55
**Thymine**	0.9935	3724146.35
**Valine**	0.9950	3981603.45

Supplementary Figures

A) 1,3-diaminopropane

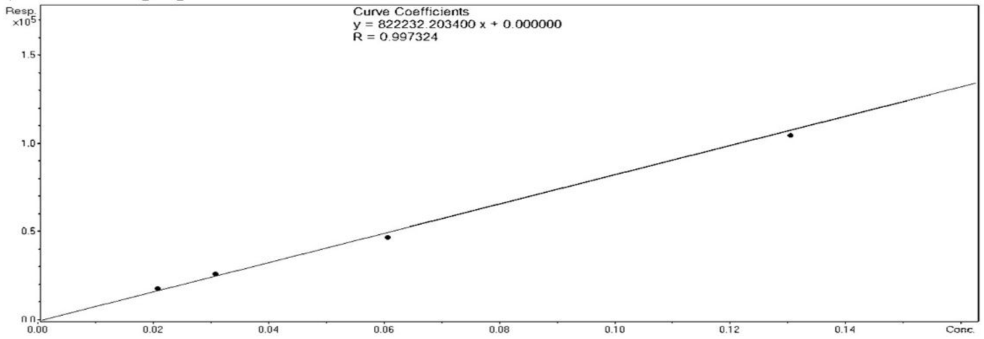

B) 2-phenylethylamine

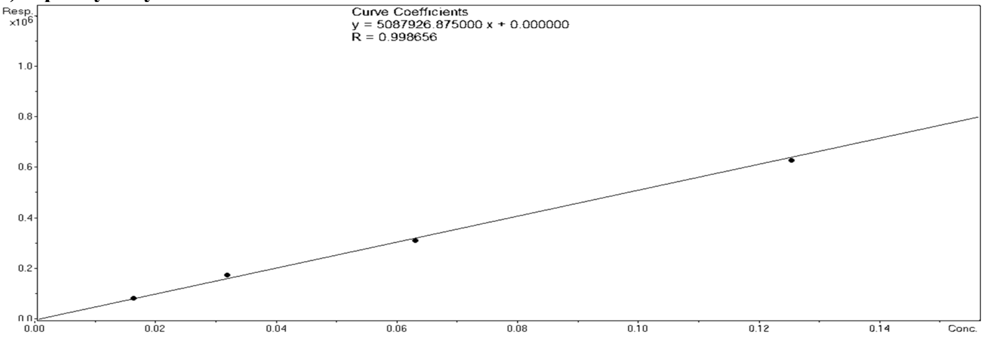

C) Aspartic acid

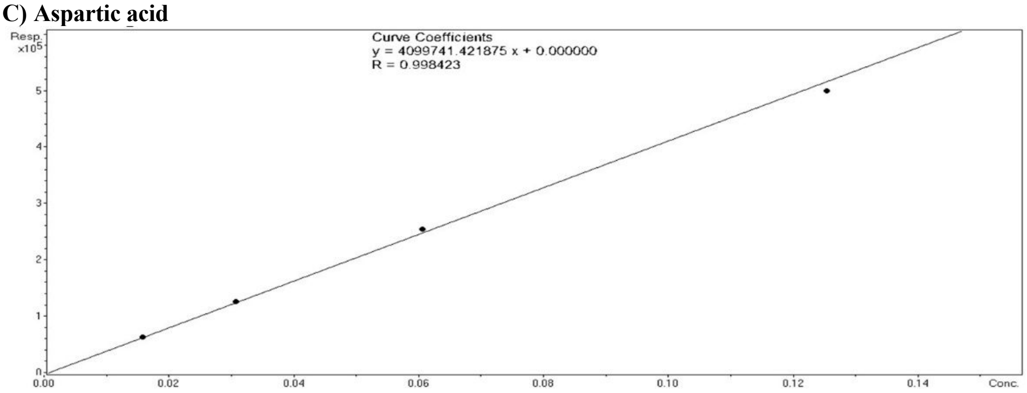

Figure S1- Calibration curves for the compounds **A**) 1,3-diaminipropoane, **B**) 2-phenylethylamine, and **C**) aspartic acid at the concentration range from 0.015 µg to 0.125 µg.

A) Glutamic acid

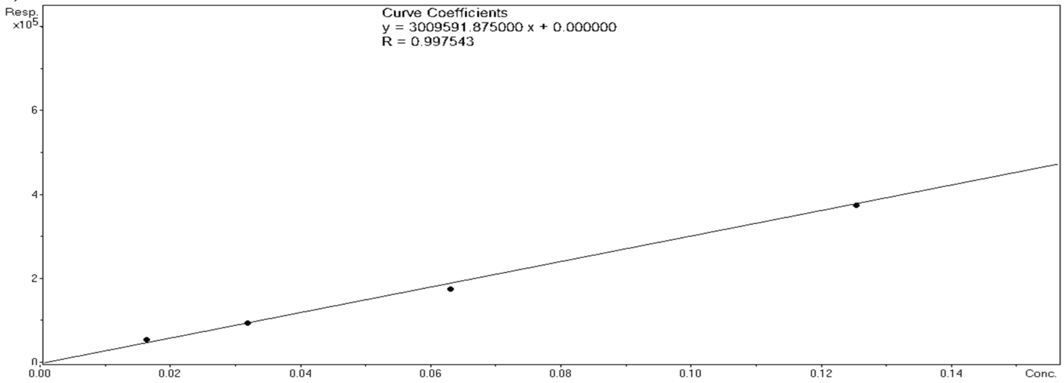

B) 3,4-di-hydroxyphenylacetic acid

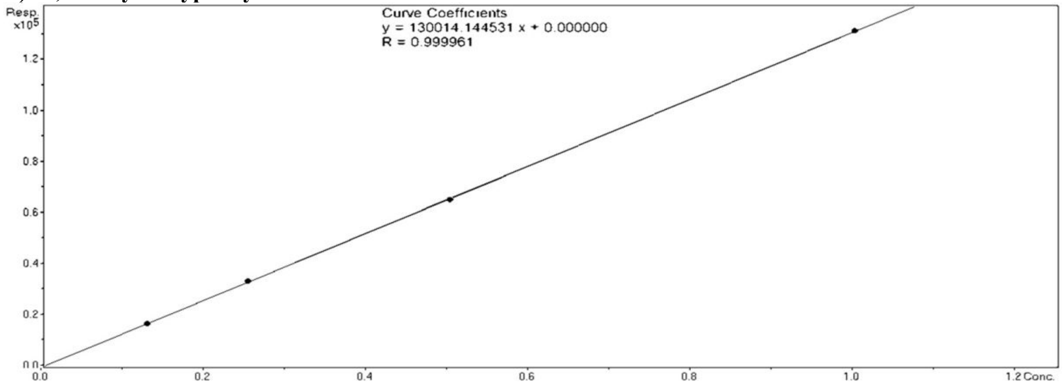

Figure S2- Calibration curves for the compounds **A**) glutamic acid, and **B**) 3,4-dihydroxyphenylacetic acid at the concentrations range from 0.015 µg to 0.125 µg.

A) 4-hydroxyphenyl acetic acid

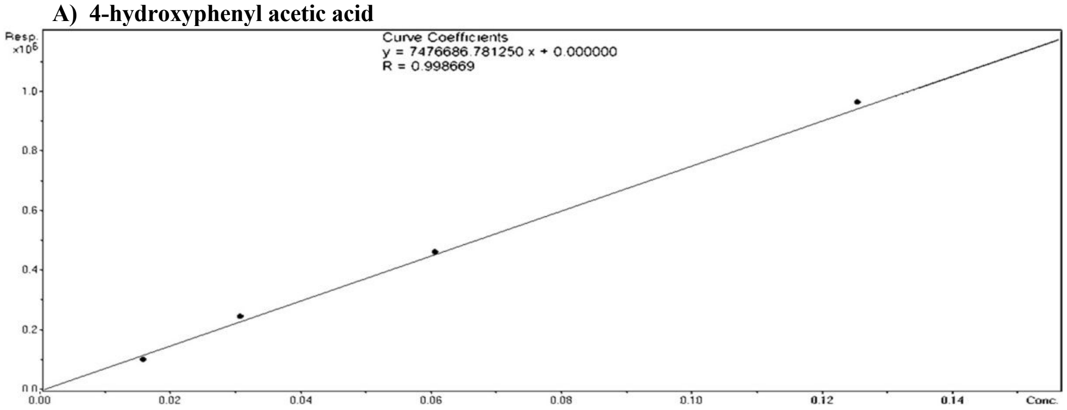

B) 5-hydroxyindoleacetic acid

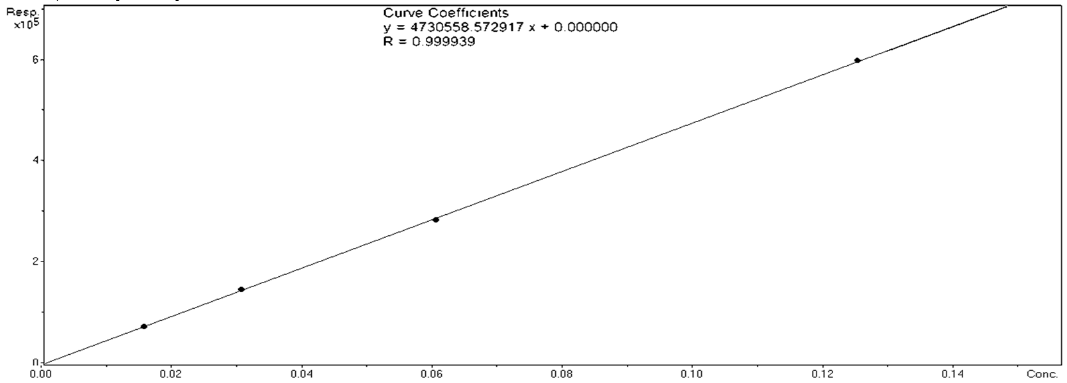

C) Indoleacetic acid

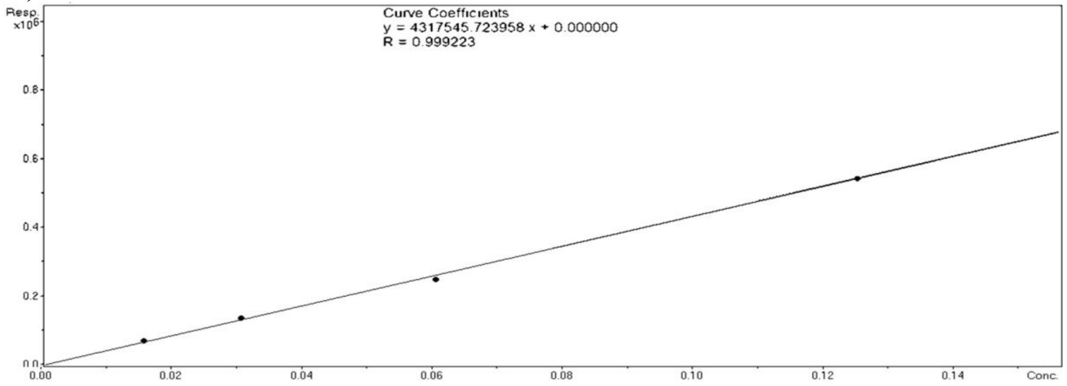

D) Kainic acid

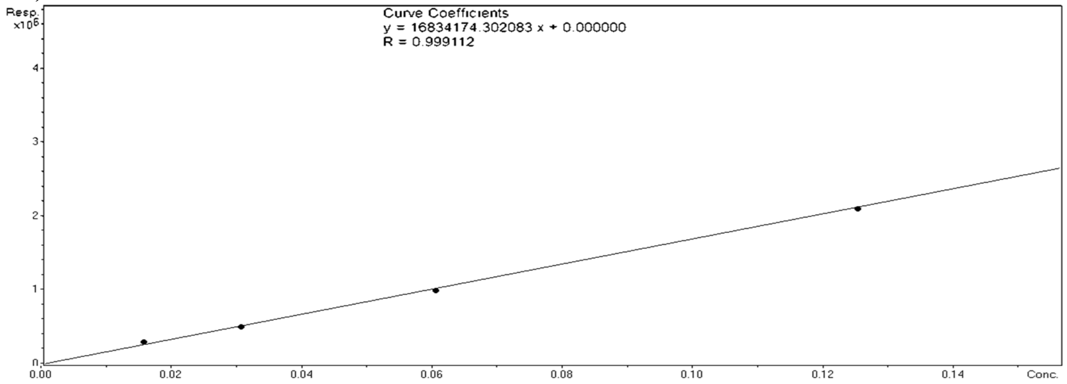

Figure S3- Calibration curves for the compounds **A)** 4-hydroxyphenylacetic acid, **B**) 5-hydroxyindoleacetic acid and **C**) indolacetic acid, and **D**) kainic acid at the concentration range from 0.015 µg to 0.125 µg.

A) Maleic acid

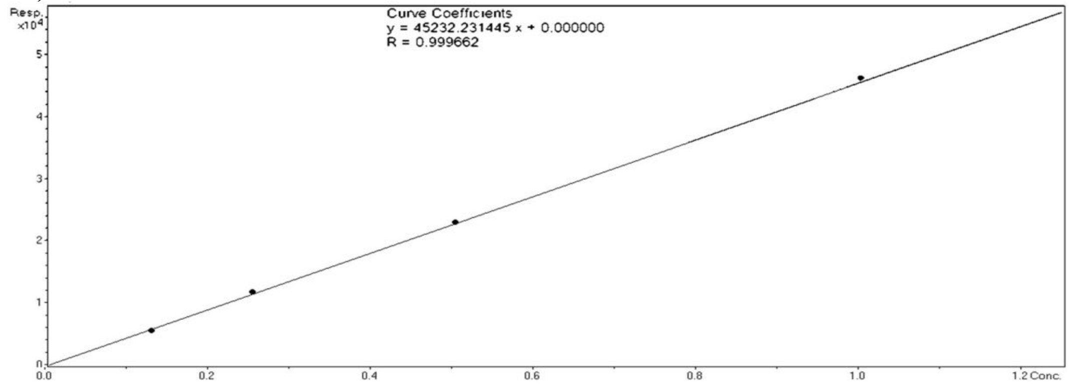

B) Adenosine diphosphate

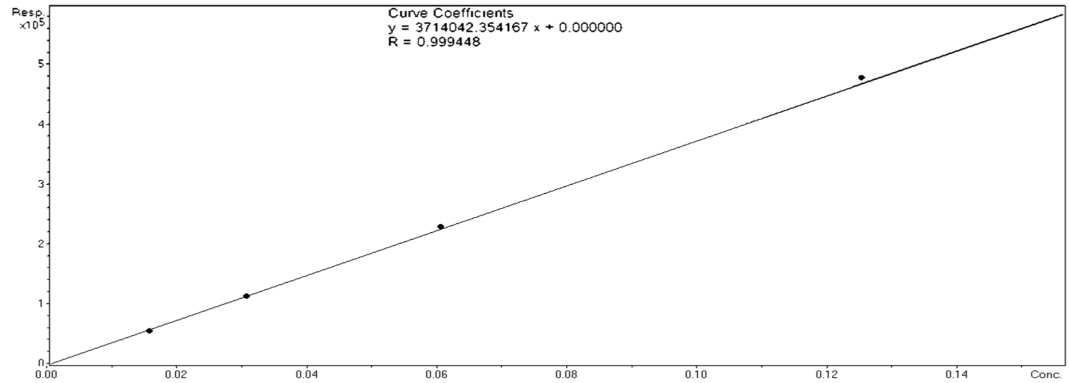

C) Adenine

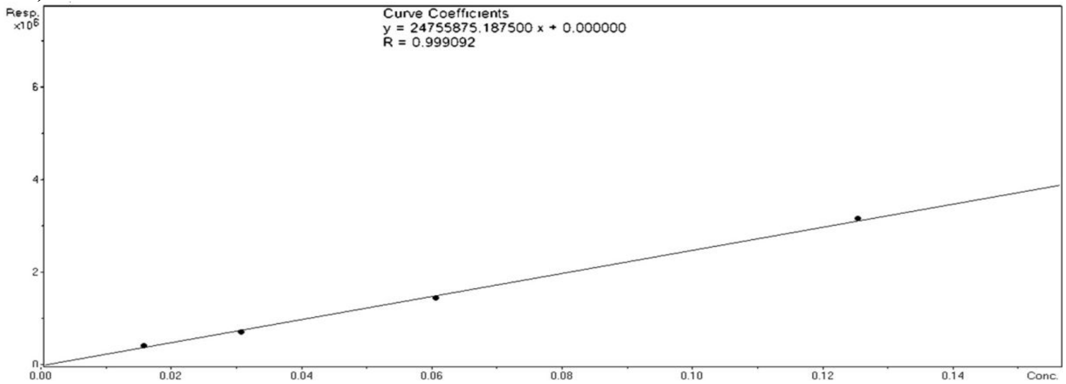

D) 3’-5’-cyclic Adenosine monophosphate

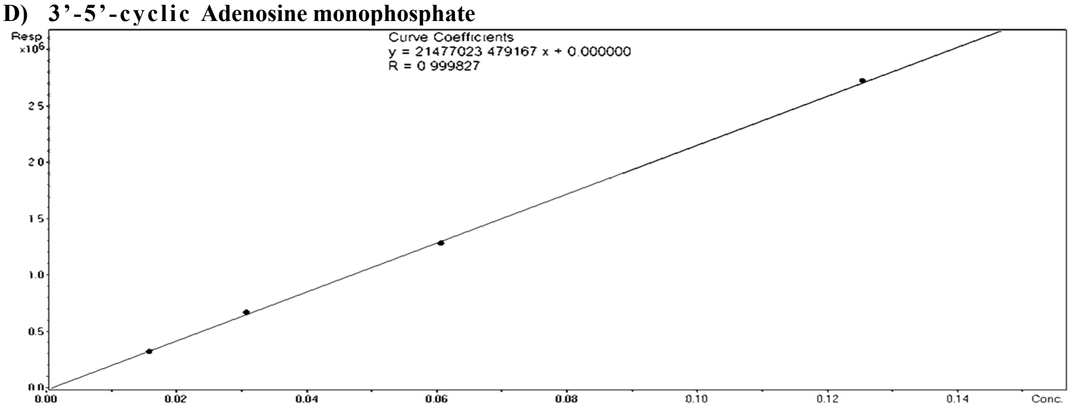

Figure S4- Calibration curves for the compounds **A)** maleic acid, **B**) adenosine diphosphate, **C**) adenine and **D**) 3’-5’-cyclic adenosine monophosphate, at the concentration range from 0.015 µg to 0.125 µg.

A) **Adenosine 5-monophosphate**

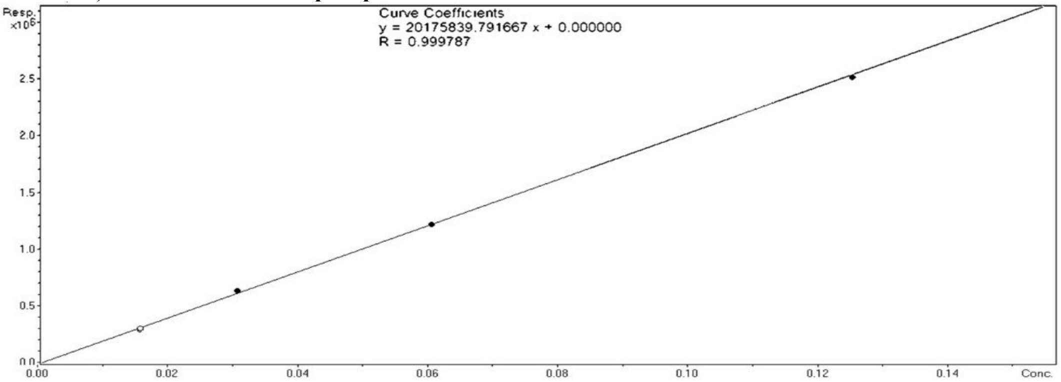

B) **Adenosine**

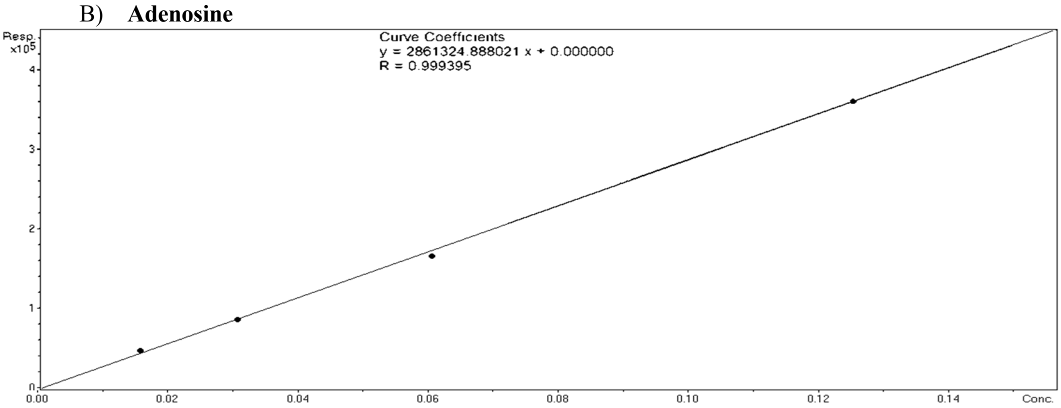

C) **Alanine**

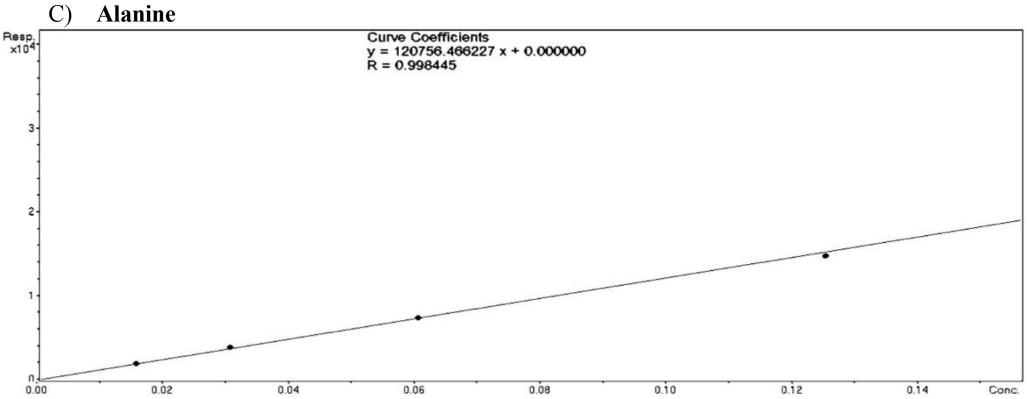

D) **Arginine**

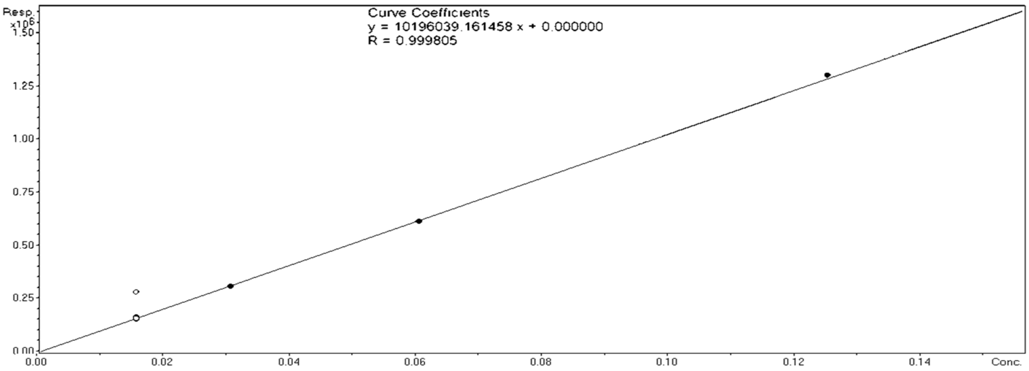

Figure S5- Calibration curves for the compounds: **A**) adenosine 5-monophosphate, **B**) adenosine, **C**) alanine, and **D**) arginine, at the concentration range from 0.015 µg to 0.125 µg.

A) Asparagine

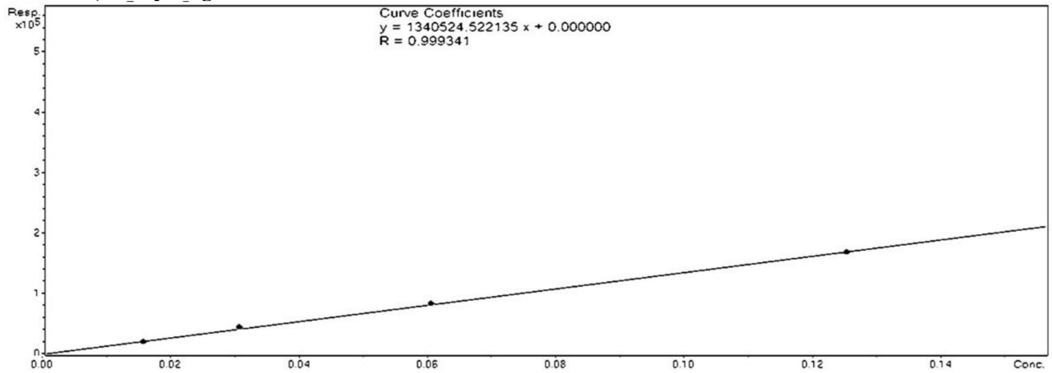

B) Betaine

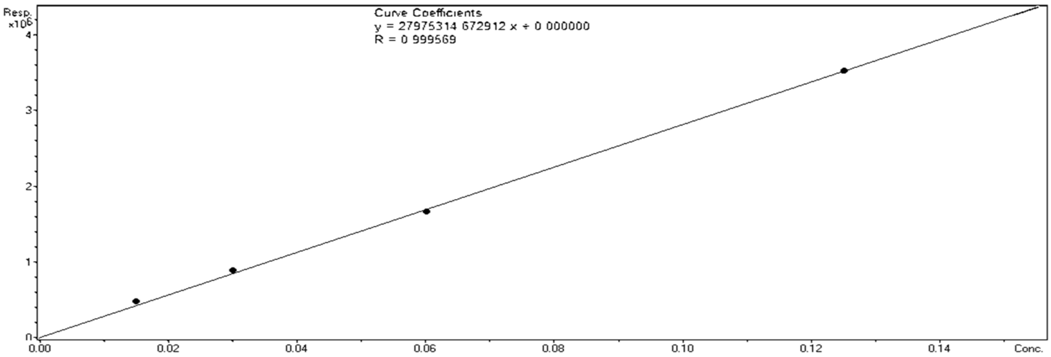

C) cadaverine

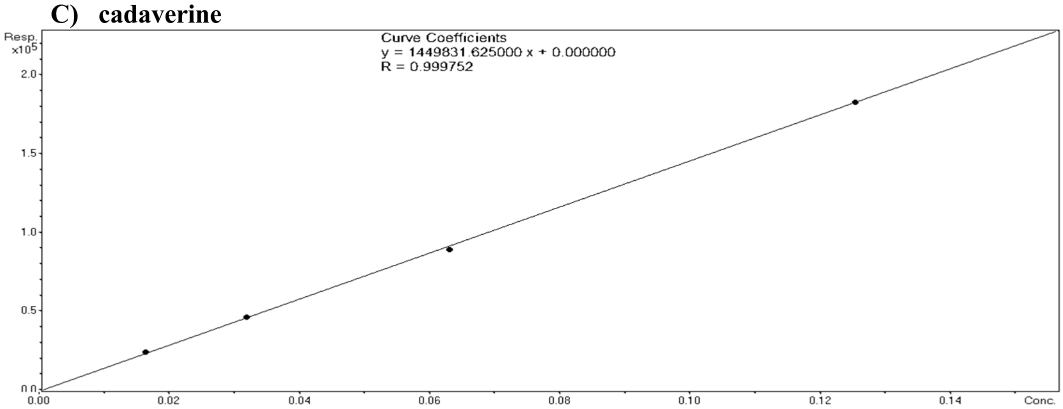

D) cytosine

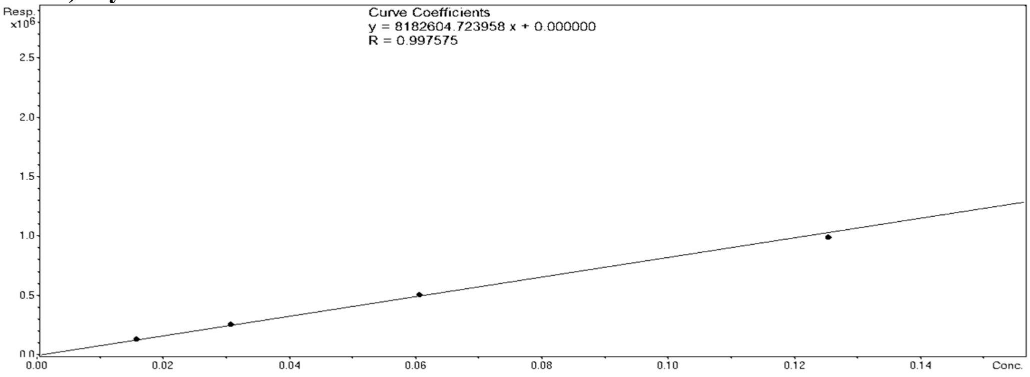

Figure S6- Calibration curves for the compounds: **A**) asparagine, **B**) betaine, **C**) cadaverine and **D**) Cytosine, at the concentration range from 0.015 µg to 0.125 µg.

A) Phenylalanine

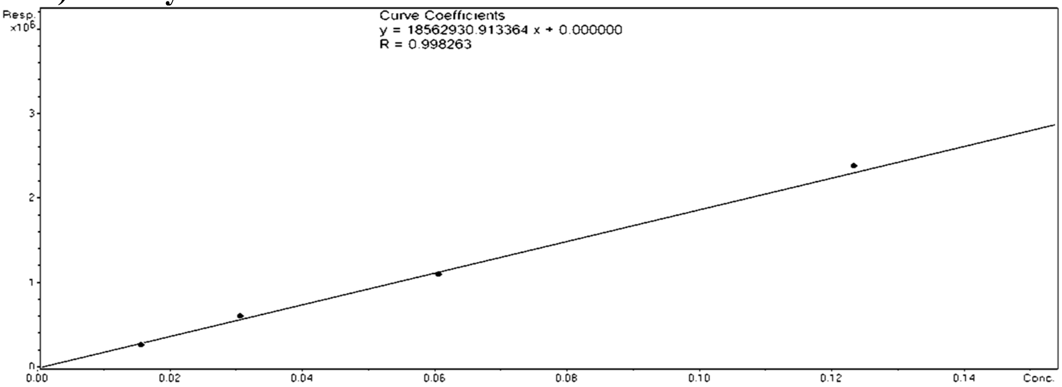

B) GABA

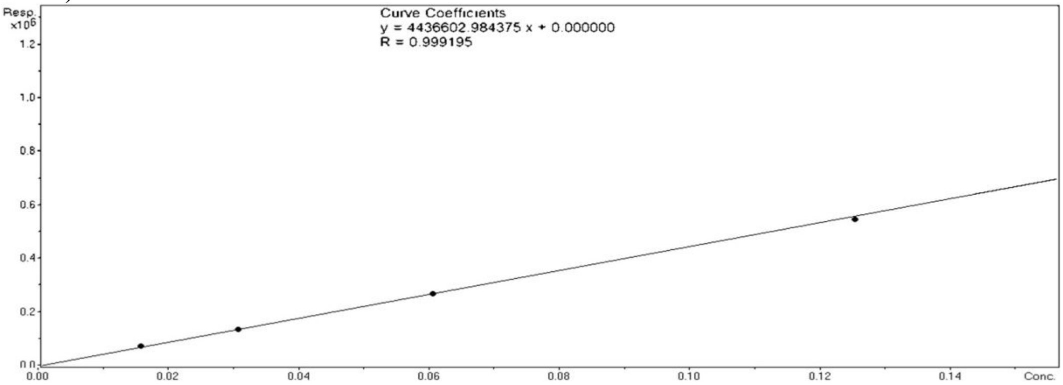

C) Glycine

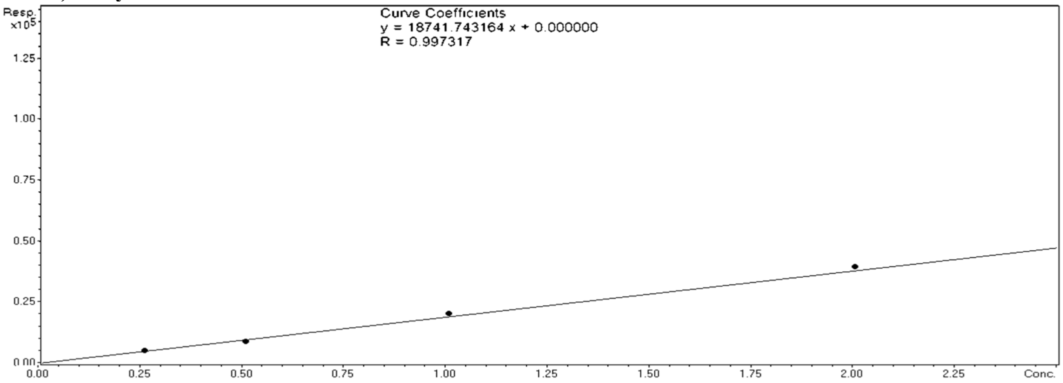

Figure S7- Calibration curves for the compounds: **A**) phenylalanine, **B)** GABA, and **C**) glycine, at the concentration range from 0.015 µg to 0.125 µg.

A) Dopamine

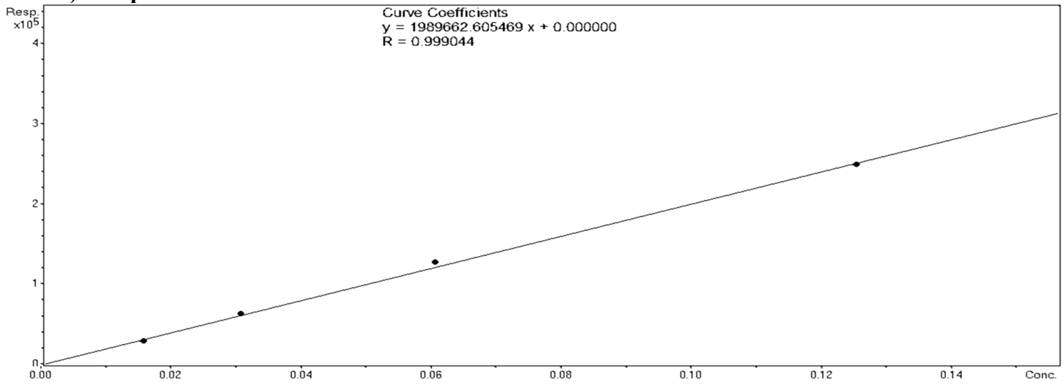

B) Epinephrine

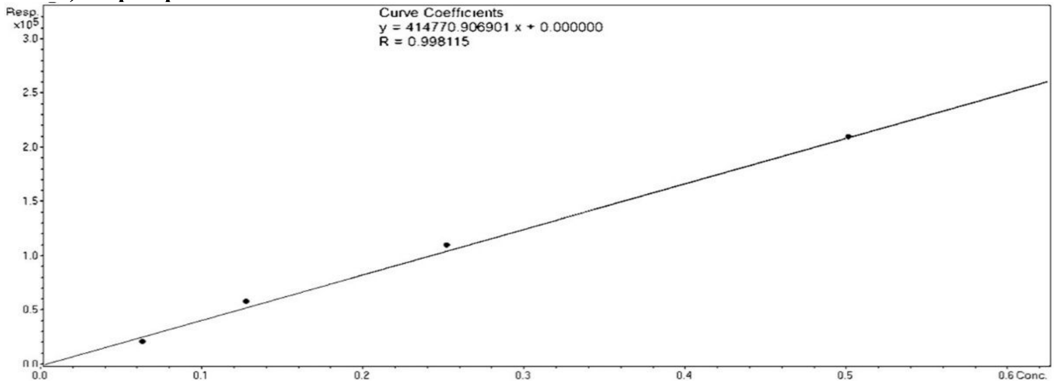

C) Spermidine

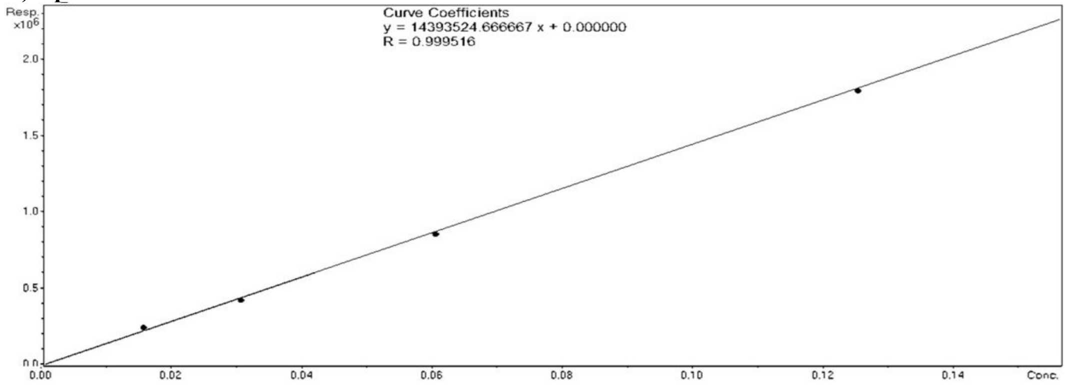

D) Spermine

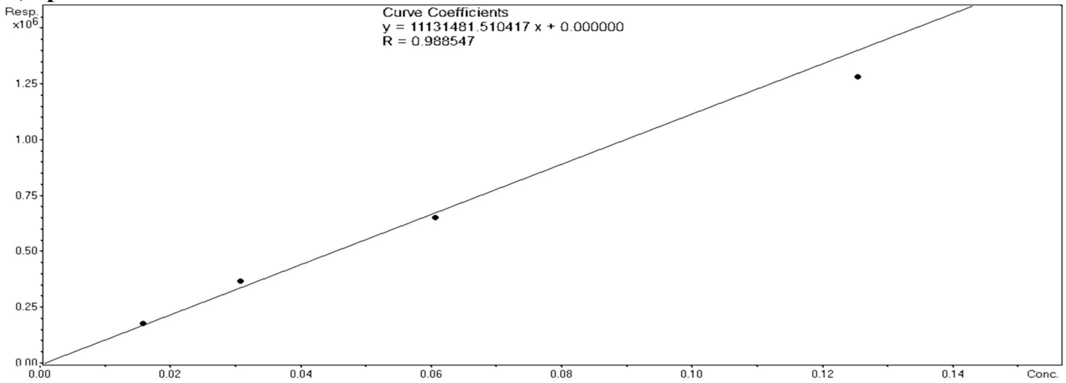

Figure S8- Calibration curves for the compounds: **A**) dopamine, **B**) epinephrine, **C**) spermidine, and **D**) spermine, at the concentration range from 0.015 µg to 0.125 µg.

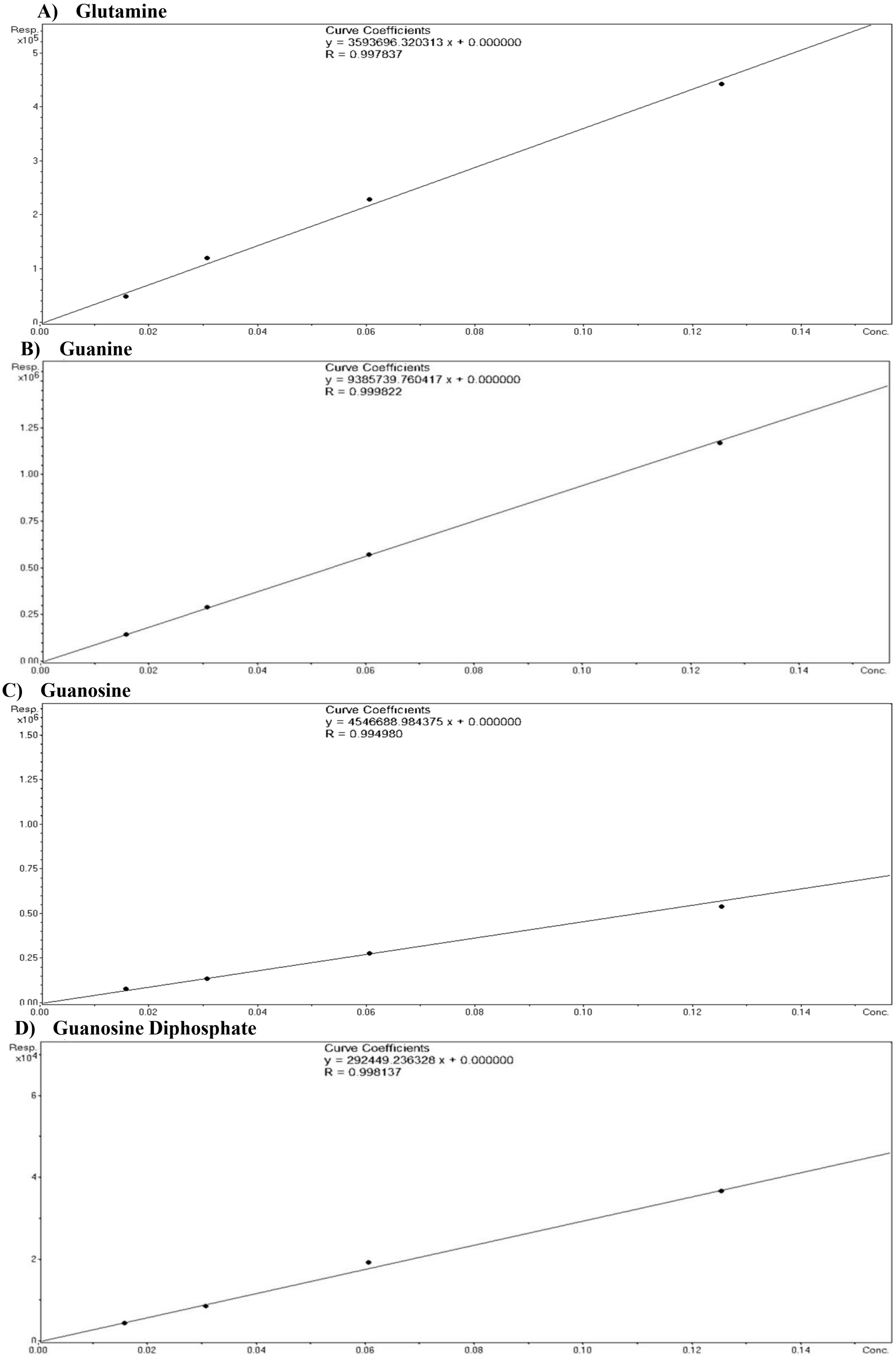

Figure S9- Calibration curves for the compounds: **A**) glutamine, **B**) guanine, **C**) guanosine, and **D**) guanosine diphosphate, at the concentration range from 0.015 µg to 0.125 µg.

A) Guanosine monophosphate

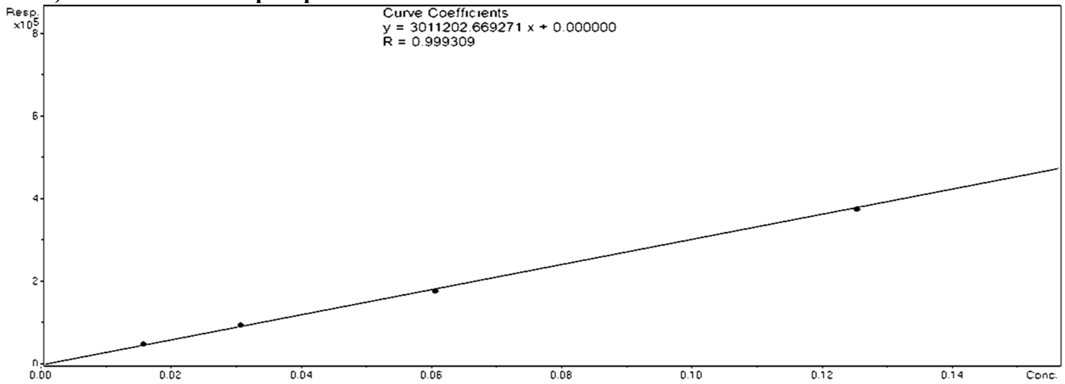

B) Hydroxytripargine

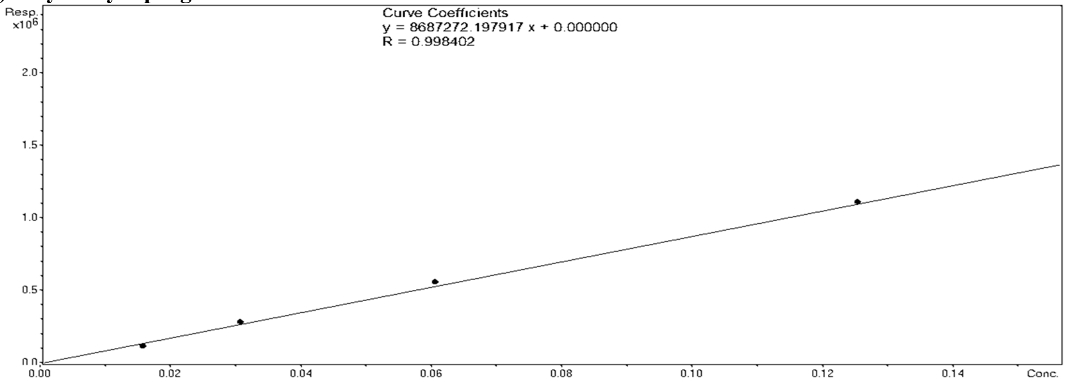

C) Histamine

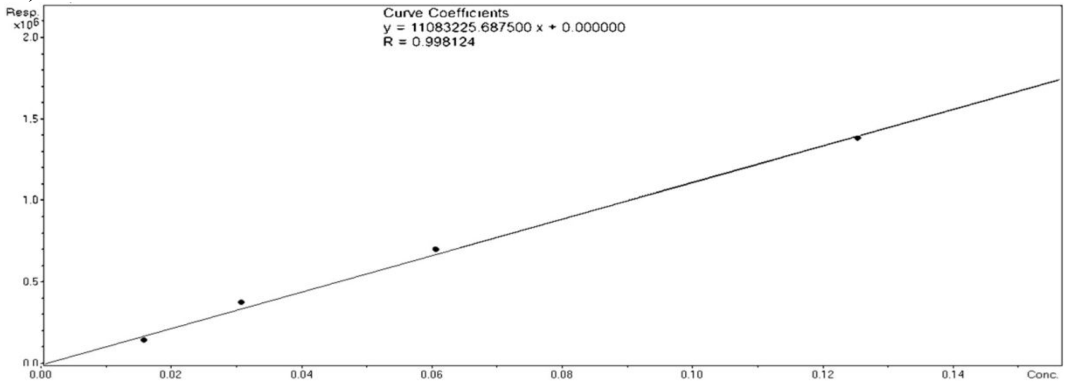

Figure S10- Calibration curves for the compounds: **A**) guanosine monophosphate, **B**) hydroxytripargine, and **C**) histamine and **D**) histamine, at the concentration range from 0.015 µg to 0.125 µg.

A) Histidine

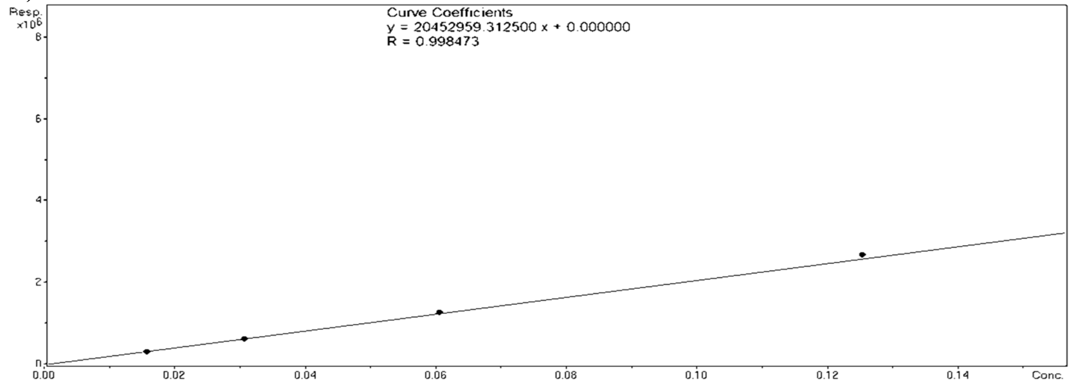

B) Hidroxyproline proline

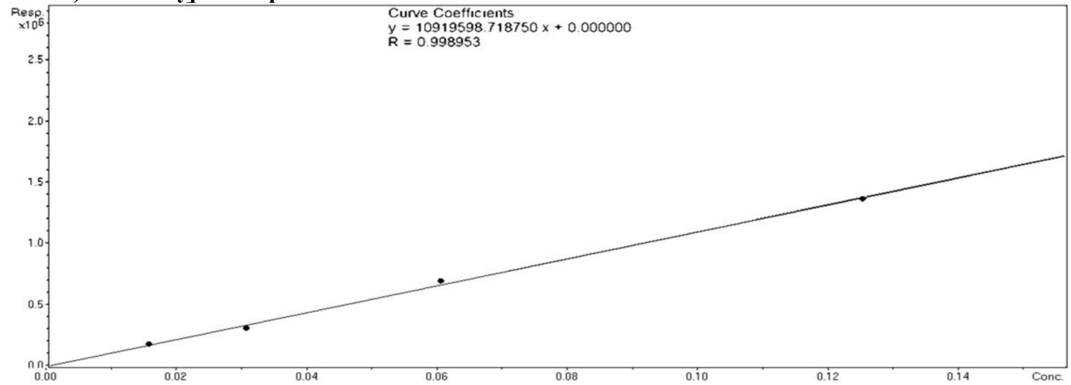

C) Isoleucine

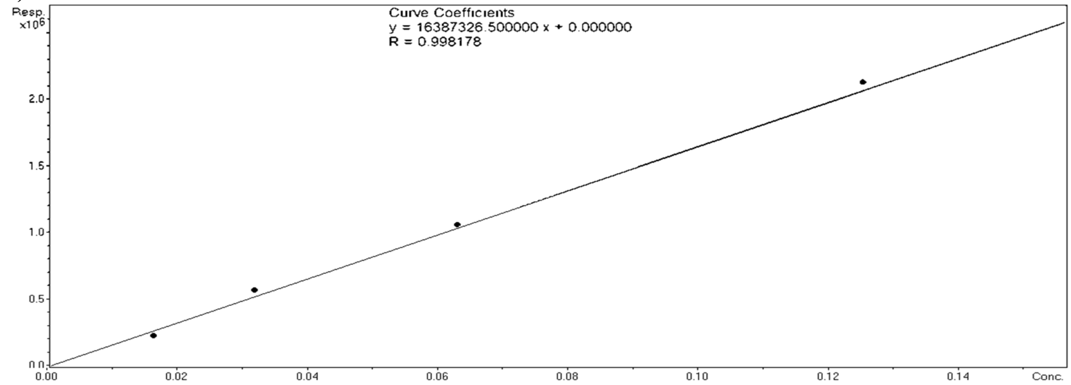

D) Leucine

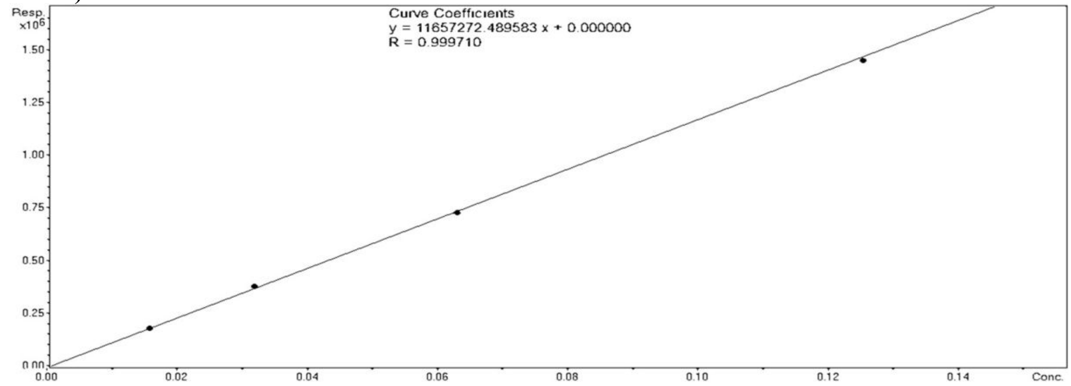

Figure S11- Calibration curves for the compounds: **A**) histidine, **B**) hydroxyproline, **C**) isoleucine, and **D**) leucine at the concentration range from 0.015 µg to 0.125 µg.

A) Lysine

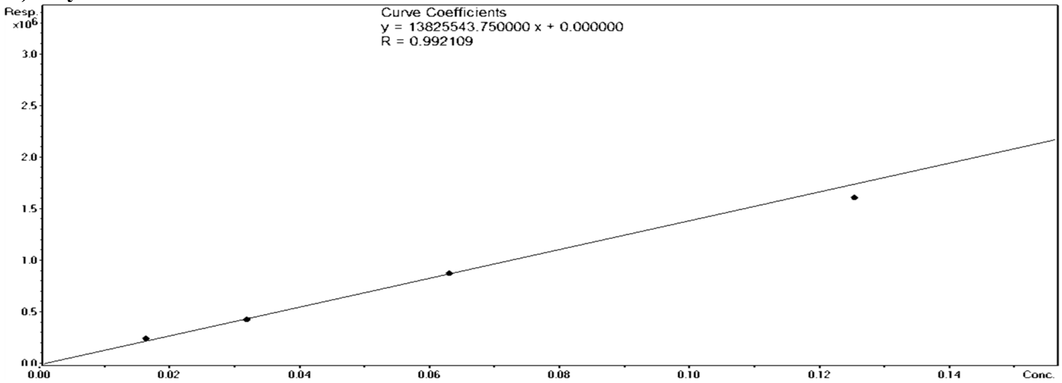

B) Methionine

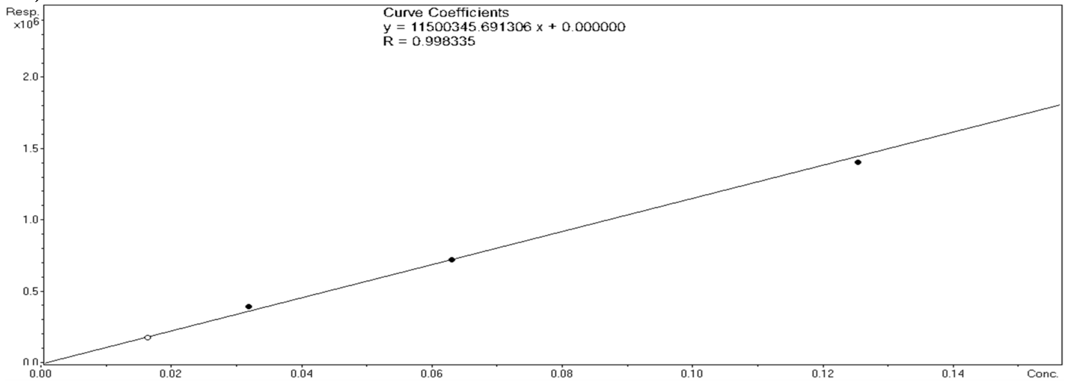

C) Nicotine

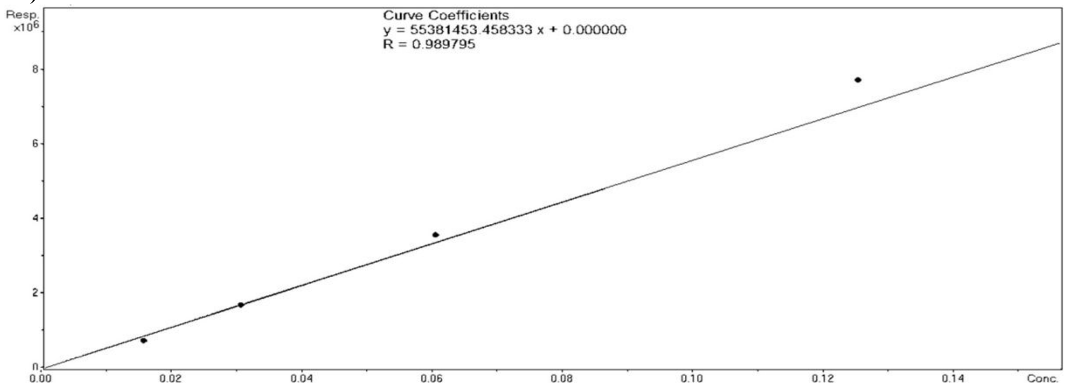

Figure S12- Calibration curves for the compounds: **A**) lysine, **B**) methionine**,** **C**) nicotine, at the concentration range from 0.015 µg to 0.125 µg.

A) Octopamine

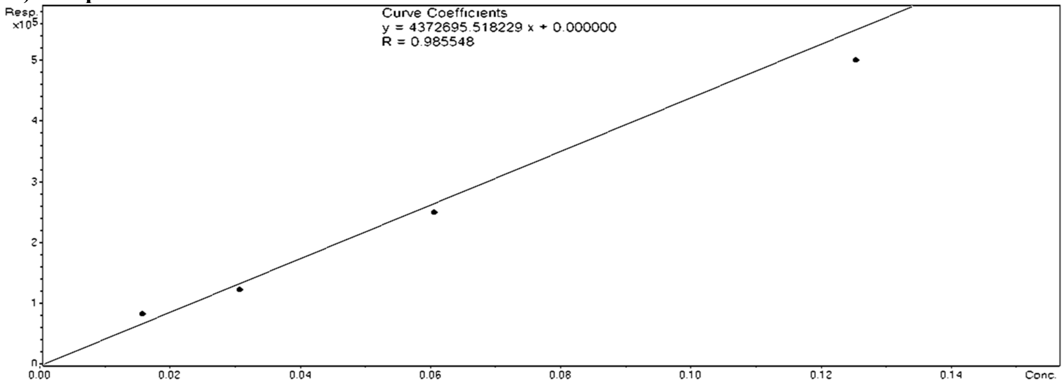

B) Proline

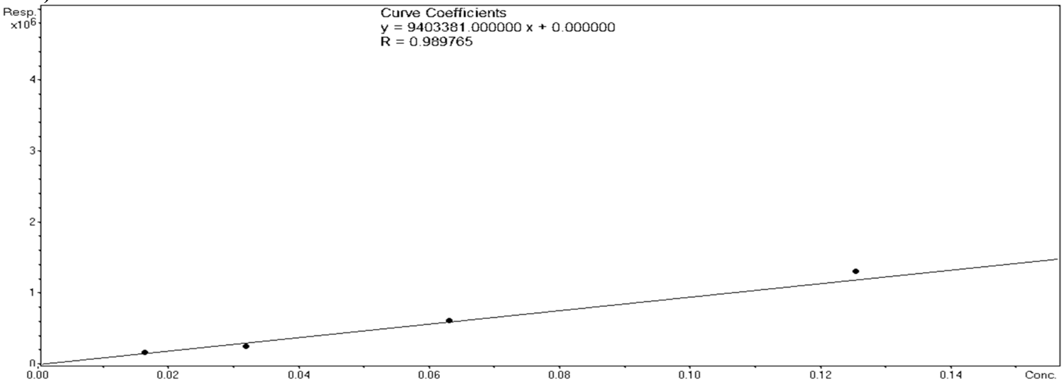

C) Putrescine

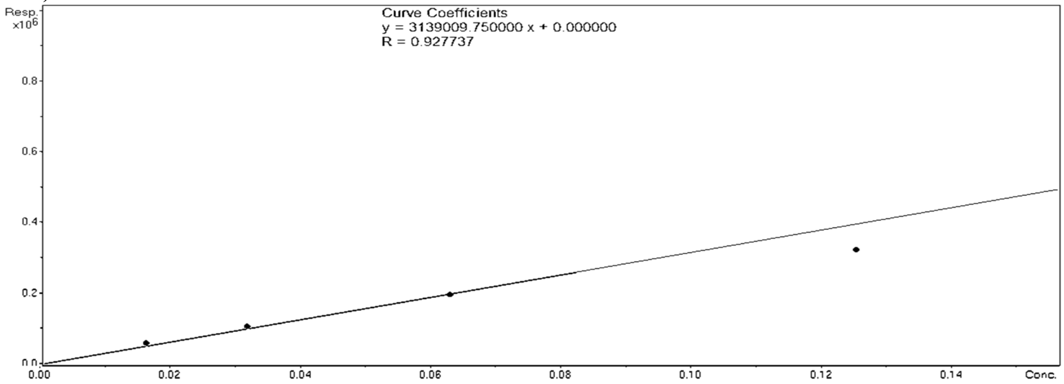

Figure S13- Calibration curves for the compounds: **A**) octopamine, **B**) proline, and **C**) putrescine, at the concentration range from 0.015 µg to 0.125 µg.

A) Serine

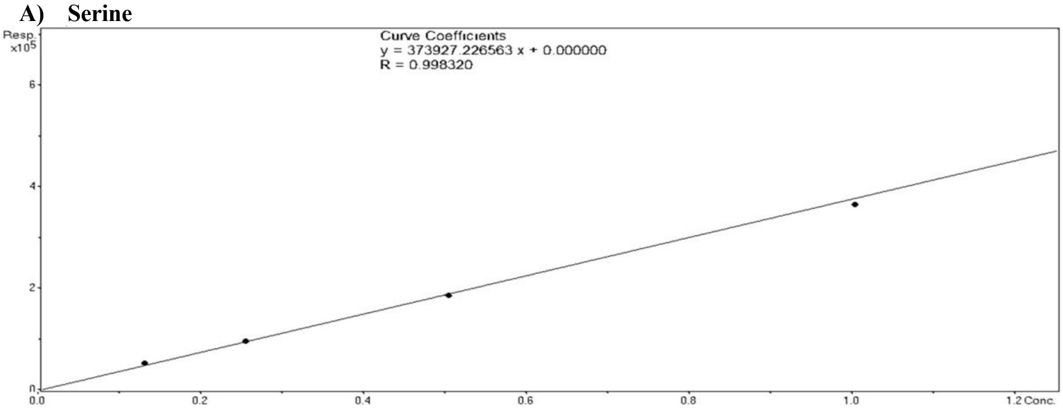

B) Serotonin

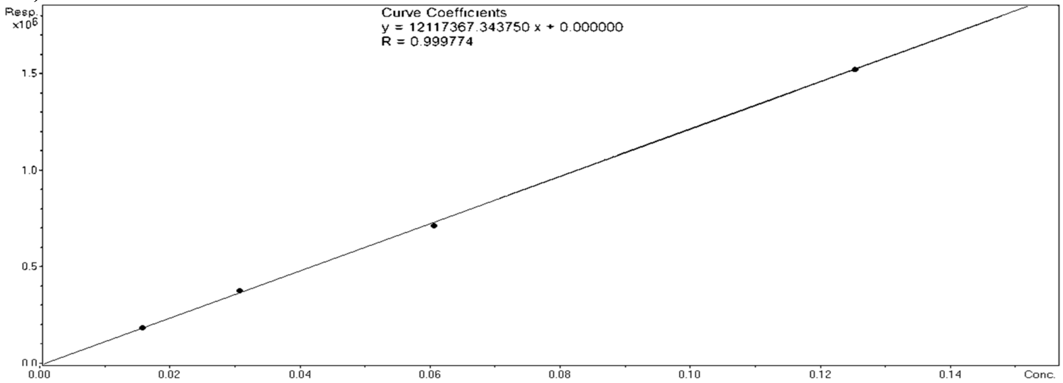

C) Taurine

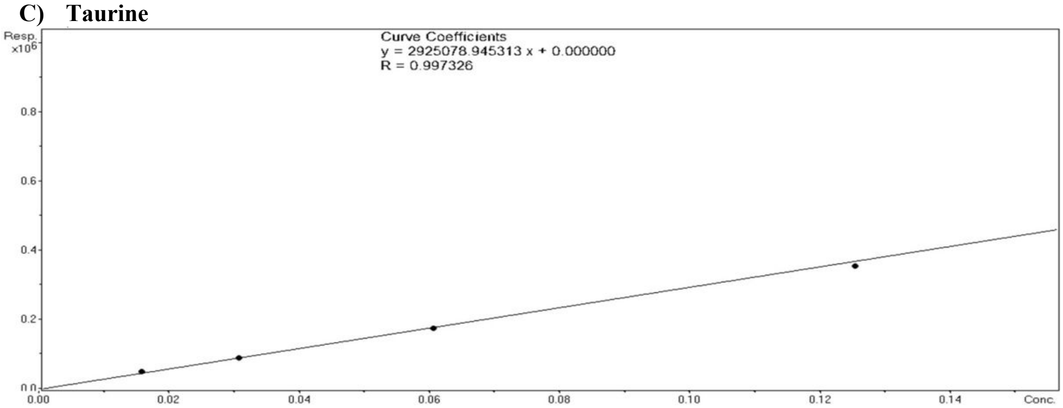

Figure S14- Calibration curves for the compounds: **A**) serine, **B)** serotonin, and **C**) taurine, at the concentration range from 0.015 µg to 0.125 µg.

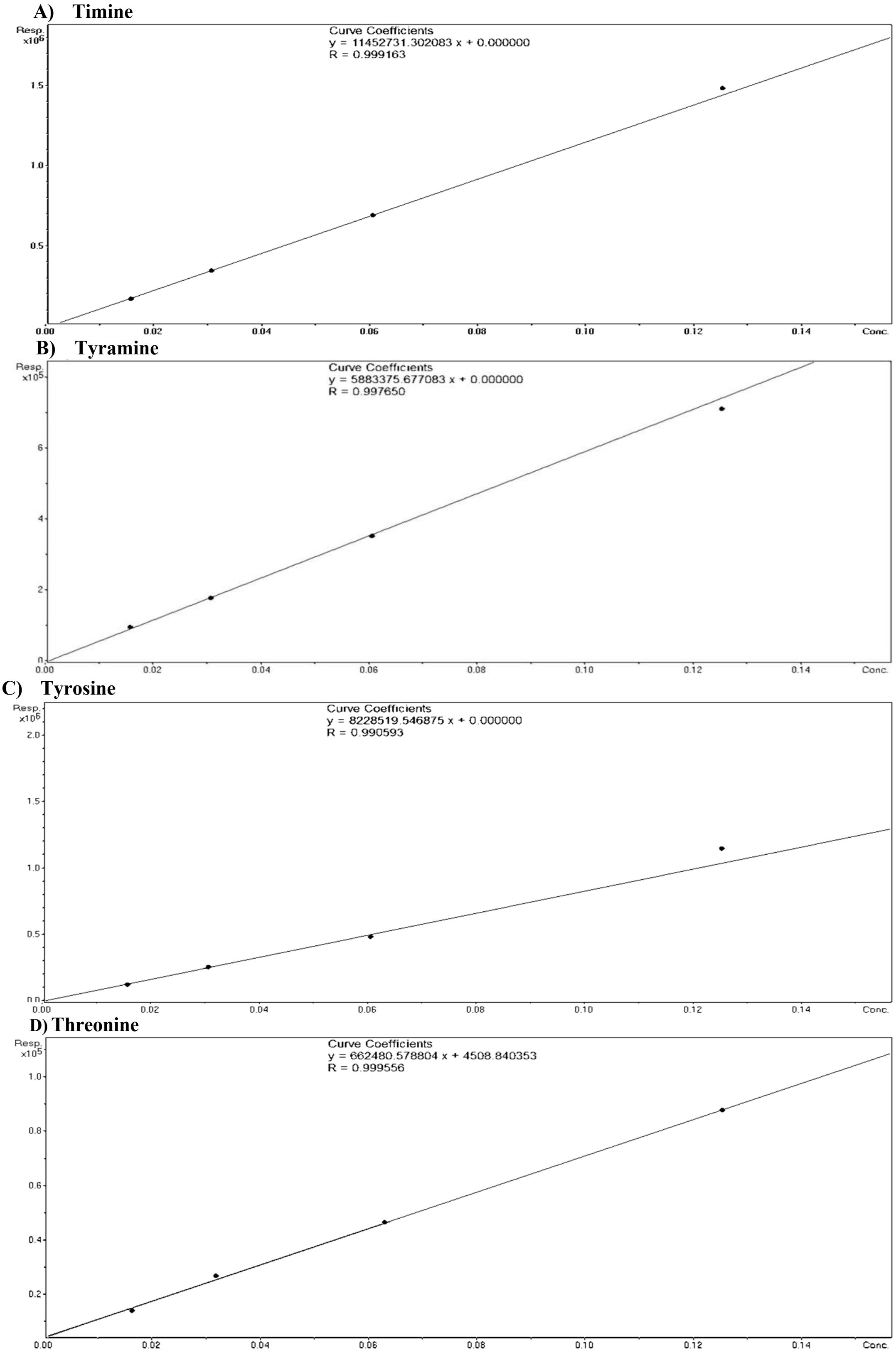

Figure S15 - Calibration curves for the compounds: **A**) timine, **B**) tyramide, **C**) tyrosine, and **D**) threonine at the concentration range from 0.015 µg to 0.125 µg.

A) Trypargine

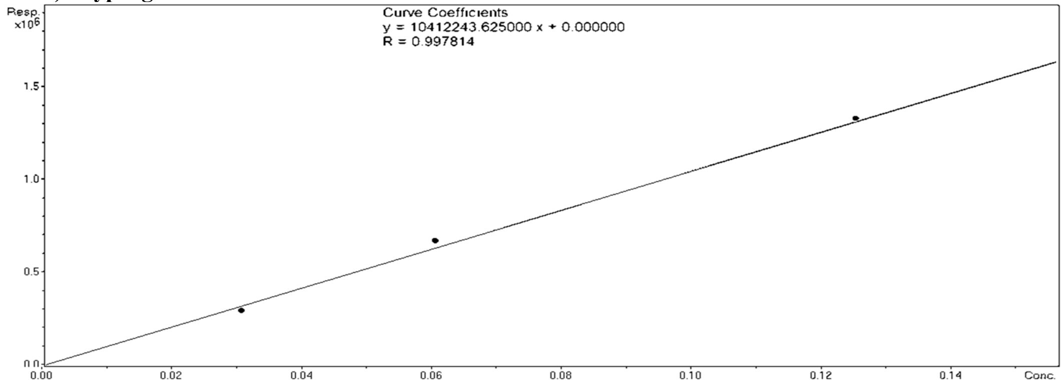

B) Tryptophan

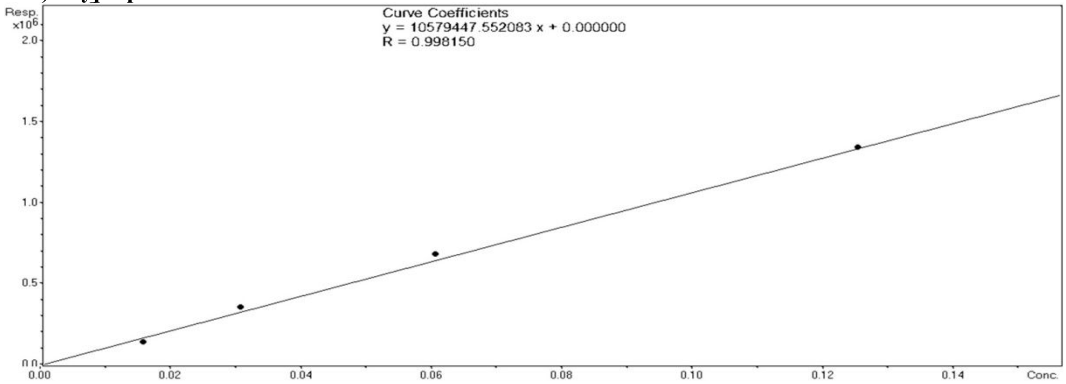

C) Uracyl

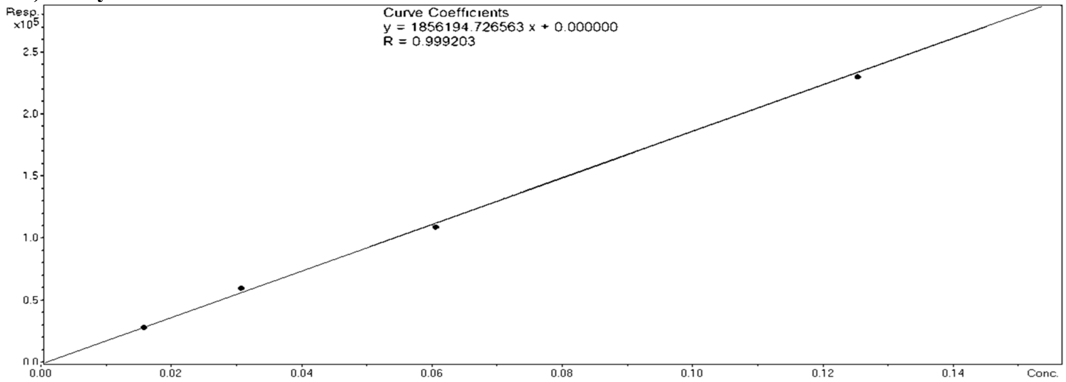

D) Valine

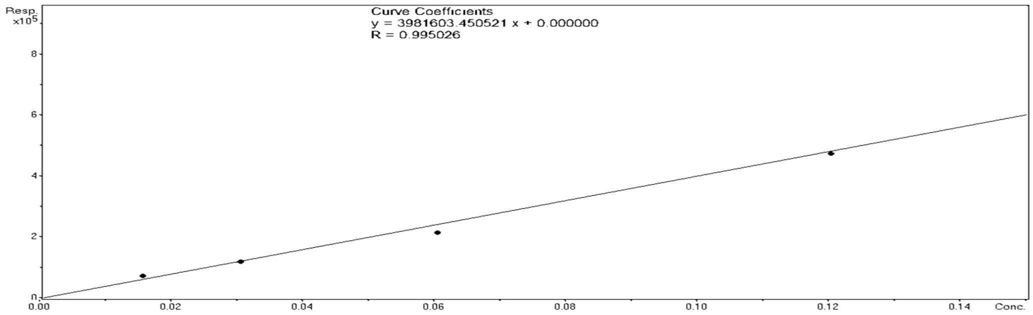

Figure S16- Calibration curves for the compounds: **A)** trypargine, **B**) tryptofan, **C**) uracyl, and **D**) valine at the concentration range from 0.015 µg to 0.125 µg.
